# Genetic Requirements for Signaling from an Autoactive Plant NB-LRR Intracellular Innate Immune Receptor

**DOI:** 10.1371/journal.pgen.1003465

**Published:** 2013-04-25

**Authors:** Melinda Roberts, Saijun Tang, Anna Stallmann, Jeffery L. Dangl, Vera Bonardi

**Affiliations:** 1 Department of Biology, University of North Carolina, Chapel Hill, North Carolina, United States of America; 2 College of Biological Sciences, China Agricultural University, Beijing, China; 3 Howard Hughes Medical Institute, University of North Carolina, Chapel Hill, North Carolina, United States of America; 4 Curriculum in Genetics and Molecular Biology, University of North Carolina, Chapel Hill, North Carolina, United States of America; 5 Department of Microbiology and Immunology, University of North Carolina, Chapel Hill, North Carolina, United States of America; 6 Carolina Center for Genome Sciences, University of North Carolina, Chapel Hill, North Carolina, United States of America; Virginia Tech, United States of America

## Abstract

Plants react to pathogen attack via recognition of, and response to, pathogen-specific molecules at the cell surface and inside the cell. Pathogen effectors (virulence factors) are monitored by intracellular nucleotide-binding leucine-rich repeat (NB-LRR) sensor proteins in plants and mammals. Here, we study the genetic requirements for defense responses of an autoactive mutant of ADR1-L2, an Arabidopsis coiled-coil (CC)-NB-LRR protein. ADR1-L2 functions upstream of salicylic acid (SA) accumulation in several defense contexts, and it can act in this context as a “helper” to transduce specific microbial activation signals from “sensor” NB-LRRs. This helper activity does not require an intact P-loop. ADR1-L2 and another of two closely related members of this small NB-LRR family are also required for propagation of unregulated runaway cell death (rcd) in an *lsd1* mutant. We demonstrate here that, in this particular context, ADR1-L2 function is P-loop dependent. We generated an autoactive missense mutation, ADR1-L2_D484V_, in a small homology motif termed MHD. Expression of ADR1-L2_D848V_ leads to dwarfed plants that exhibit increased disease resistance and constitutively high SA levels. The morphological phenotype also requires an intact P-loop, suggesting that these ADR1-L2_D484V_ phenotypes reflect canonical activation of this NB-LRR protein. We used ADR1-L2_D484V_ to define genetic requirements for signaling. Signaling from ADR1-L2_D484V_ does not require NADPH oxidase and is negatively regulated by *EDS1* and *AtMC1*. Transcriptional regulation of *ADR1-L2_D484V_* is correlated with its phenotypic outputs; these outputs are both SA–dependent and –independent. The genetic requirements for ADR1-L2_D484V_ activity resemble those that regulate an SA–gradient-dependent signal amplification of defense and cell death signaling initially observed in the absence of LSD1. Importantly, *ADR1-L2_D484V_* autoactivation signaling is controlled by both *EDS1* and SA in separable, but linked pathways. These data allows us to propose a genetic model that provides insight into an SA–dependent feedback regulation loop, which, surprisingly, includes ADR1-L2.

## Author Summary

Plants possess an active, inducible disease resistance system, and induction of these responses depends in part on plant resistance proteins. Present understanding of these resistance proteins likens them to molecular switches that bind nucleotides to activate disease resistance responses. Previously it was shown that Activated Disease Resistance 1-like 2 (ADR1-L2), a plant disease resistance protein, is important in the immune response, but can function in the contexts analysed independently of what is currently considered the canonical nucleotide switch activation. Here, we show that, in addition to these previously reported functions, ADR1-L2 also works as a typical, activated disease resistance protein. We use an autoactive mutant form of the protein and show that it promotes disease resistance. We find that ADR1-L2 works in an EDS1-dependent feedback loop with salicylic acid, a hormone known to be essential for plant disease resistance. This work allows us to broaden the understanding of how plant disease resistance proteins function to generate defense against pathogens.

## Introduction

Plants encounter a wide variety of pathogens. To defend against infection, plants rely on their organ surfaces as pre-formed barriers to infection. Plants have also evolved an active, two-layered immune system [1]. The first branch utilizes transmembrane receptors (PRRs, or pattern recognition receptors) which detect microbe-associated molecular patterns (MAMPs) of various pathogens [2]. MAMP detection elicits a rapid, relatively low-amplitude host transcriptional response resulting in MAMP-triggered immunity (MTI) which is sufficient to halt growth of many microbes [1], [3]. Successful pathogens can suppress or delay MTI via delivery of effector molecules into host cells. Effectors are typically virulence proteins [4]. Gram-negative bacterial pathogens deliver effectors via injection into the plant cell by the Type III Secretion System (TTSS). Plants respond to effectors with the second tier of recognition, which is dependent on highly polymorphic intracellular disease resistance (R) proteins of the NB-LRR family. NB-LRRs are specifically activated by the presence and/or action of effectors to trigger robust defense responses termed Effector-Triggered Immunity (ETI), which can include localized hypersensitive cell death [1].

NB-LRR proteins are members of the signal transduction ATPases with numerous domains (STAND) superfamily, which also includes animal innate immune sensors of the nucleotide-binding domain and leucine-rich repeat-containing (NLR) class [5], [6]. STAND proteins are ATPases that function as molecular switches: in the “off” position they bind ADP, and in the “on” position they bind ATP, activating nucleotide hydrolysis and triggering downstream defense responses. This model is proposed for plant NB-LRRs, though there is very little experimental data pertinent to it [7]. Two essential, conserved homology regions necessary for proper plant NB-LRR activity are the P-loop (Walker-A) and the thus far plant-specific ‘MHD motif’ located in the ARC2 sub-domain of the extended NB-ARC domain. Mutations in the P-loop typically lead to loss of function [8], [9]. Conversely, mutation of the Asp (D) in the MHD motif often leads to autoactivity of the NB-LRR protein [10]–[15], resulting in either lethality or a severely dwarfed morphology. These pleiotropic phenotypes are thought to be the consequence of ectopic accumulation of SA, a key defense hormone whose synthesis from chorismate is controlled by the isochorismate synthase gene (ICS1/SID2) [16], and consequent defense activation [11], [13], [15]. Additionally, several NB-LRRs, in both plants and animals, work in pairs: in these cases, one can function as an effector-specific ‘sensor’, and the other as a ‘helper’ protein. This may allow or drive the formation of higher-order protein complexes necessary for properly regulated defense activation [17]–[20].

ADR1-L2 (Activated Disease Resistance 1-like 2) is one of a small family of NB-LRR proteins that includes ADR1 and ADR1-L1 [21]. We recently demonstrated that ADR1-L2 functions downstream of the production of reactive oxygen intermediates (ROI), and upstream of SA accumulation, in basal defense (defined as the response that limits the growth and proliferation of genetically virulent pathogens). ADR1-L2 also functions in MAMP-triggered SA accumulation, and as a ‘helper’ protein during some, but not all ETI responses driven by effector-mediated activation of specific sensor NB-LRR proteins [22].

Surprisingly, none of the ADR1-L2 functions above required an intact P-loop [22]. In addition to these ‘non-canonical’ activities, we suggested that ADR1-L2 might have as yet undefined P-loop dependent, ‘canonical’ functions that, in the absence of the specific effector required for activation, are difficult to define. ADR1-L2 would not be the first NB-LRR protein to have multiple, independent functions. The mouse NLR protein NLRC4 has two separate functions as a ‘helper’ protein in the recognition of both the MAMP flagellin and PrgJ, a component of the Salmonella TTSS. These activities are downstream of the activation of two different sensor NLRs: NAIP5 is necessary for flagellin perception, and NAIP2 is required for PrgJ recognition [17], [20]. Importantly, NLRC4 ‘helper’ activity is also P-loop independent [17], [20].

Canonical, effector-driven NB-LRR activation typically leads to an NADPH oxidase-dependent ROI burst [23]. The *adr1* family triple mutant (*adr1 adr1-L1 adr1-L2*) exhibited normal ROI production after successful pathogen recognition [22]. Thus, the ADR1-L2 helper function noted above is downstream or independent of this oxidative burst. However, *adr1* triple mutants failed to accumulate wild-type levels of SA in this context [22]. Another protein that functions downstream of the effector-driven oxidative burst and both regulates and responds to SA accumulation is *L*esion *S*imulating *D*isease resistance 1 (LSD1) [23], [24]. Loss of LSD1 leads to improper regulation of runaway cell death, or rcd [24] that eventually engulfs the affected leaf. The Arabidopsis NADPH oxidase AtRbohD, which is required for effector-driven oxidative burst, is not required for *lsd1*-mediated cell death [23]. On the other hand, *lsd1* rcd is both induced by, and requires, SA [24], [25]. *lsd1* rcd is also regulated by Enhanced Disease Susceptibility 1 (EDS1) and a type I metacaspase, AtMC1; *eds1 lsd1* and *atmc1 lsd1* plants do not exhibit rcd [26], [27]. EDS1 is a defense response regulator, required for both basal defense and Toll/interleukin-1 (TIR)-NB-LRR mediated ETI [28]. EDS1 and SA act in a regulatory feedback loop, with SA up-regulating EDS1 expression and EDS1 functioning as a potentiator of SA-mediated signaling [29], [30]. AtMC1 is a positive regulator of ETI-mediated cell death [27].

To define the genetic requirements of putative canonical functions of ADR1-L2 in the absence of an effector known to activate it, we created an autoactive MHD mutant, *ADR1-L2_D484V_*. This allele displayed the dwarfed morphology that is the hallmark of MHD mutants [11], [13], [15]. We demonstrate that this autoactivity is P-loop dependent, downstream of AtRbohD-mediated ROI production, partially dependent on SA synthesis, and negatively regulated by EDS1 and AtMC1. We then present and validate a model for the interaction of EDS1, LSD1, and ADR1-L2, showing that these proteins function in both SA-dependent and SA-independent feedback regulatory loops that are interconnected.

## Results

### Members of the ADR1 family of NB-LRRs are required for runaway cell death in *lsd1*

**Figure 1:**
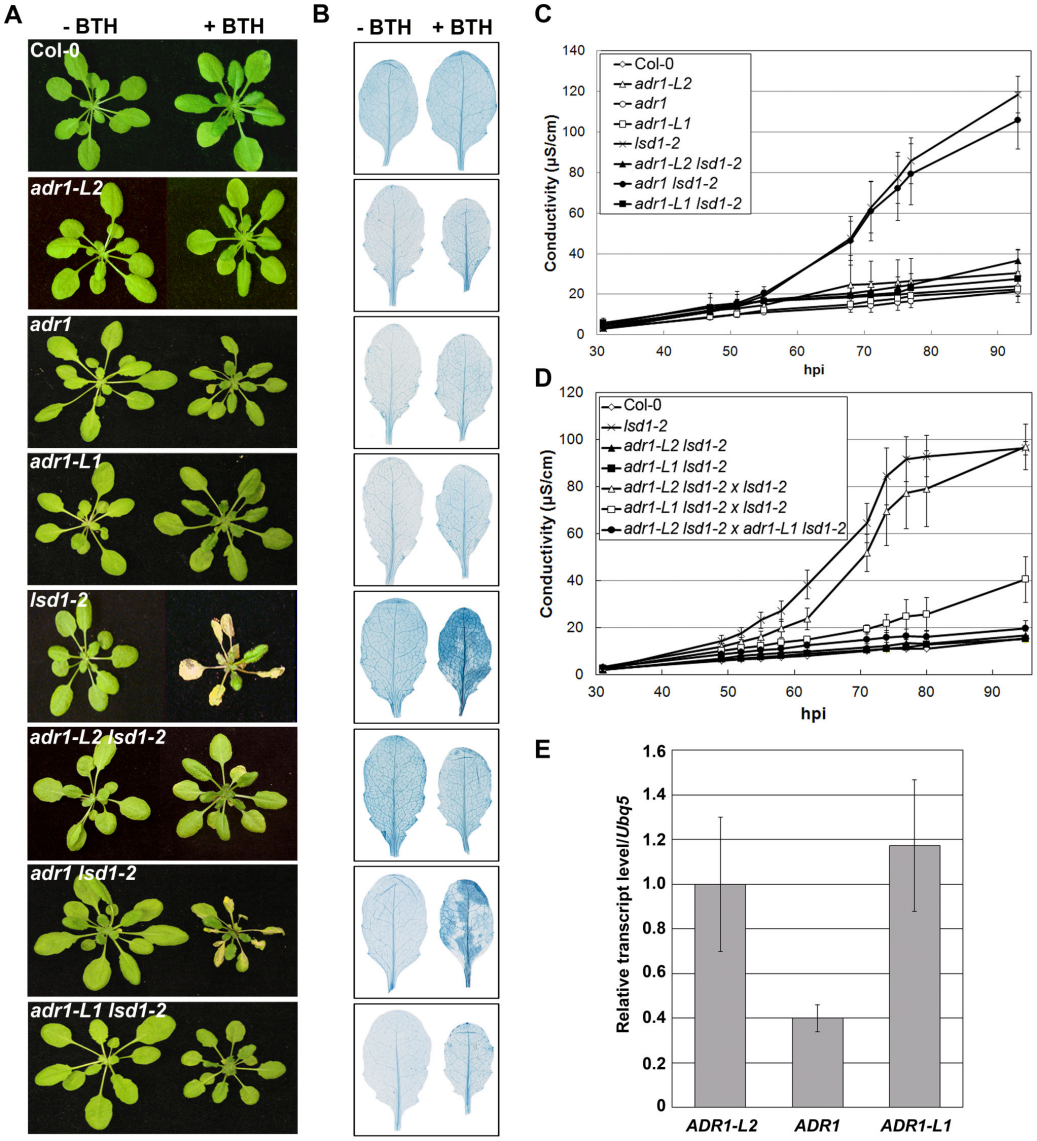
A family of CC-NB-LRR proteins is required for *lsd1* runaway cell death. (A) Four-week-old plants were sprayed with 300 µM BTH or water. Pictures of plants were taken 5 days post-inoculation (dpi). (B) Leaves from plants in (A) were stained with trypan blue to visualize cell death. Leaves on the left are water-treated controls, leaves on the right are sprayed with 300 µM BTH. (C) Ion leakage measurements from (A), 5 days post-BTH treatment. Values are means ±2× SE (n = 5). (D) Ion leakage measurements for NANC. *adr1-L1 lsd1-2*×*lsd1-2*, *adr1-L2 lsd1-2*×*lsd1-2*, *adr1-L1 lsd1-2*×*adr1-L2 lsd1-2* represent F1 plants of the indicated crosses, and are thus *lsd1* homozygous and heterozygous for the indicated *adr* mutations. (E) Quantitative real time PCR for the transcript amounts of the three members of the *ADR* family in wild-type Col-0 plants, normalized to *UBQ5*.

ADR1-L2 is a CC-NB-LRR that is a positive regulator of *lsd1* rcd [22]. It is part of a small family of NB-LRRs that includes ADR1 and ADR1-L1 [21], [22]. We generated *adr1 lsd1-2* and *adr1-L1 lsd1-2* double mutants and sprayed them with the SA analog benzothiadiazole (BTH) [31] to test whether *adr1* and *adr1-L1* also suppress the initiation and propagation of *lsd1* rcd. Col-0 wild-type plants were unaffected by BTH treatment, whereas *lsd1-2* plants sprayed with BTH showed typical rcd [24]. As reported, the *adr1-L2 lsd1-2* double mutants fully suppressed *lsd1* rcd [22]. *adr1-L1* also fully suppressed *lsd1-2* rcd, while *adr1* had only a slight effect (Figure 1A, 1B). We quantified this phenotype by monitoring cellular ion leakage via changes in media conductivity, an established proxy for membrane damage associated with cell death [32]. Col-0 plants did not exhibit significant changes in media conductivity, but *lsd1-2* plants showed increasing conductivity, with the highest reading at 92 hours post-BTH treatment. *adr1-L1 lsd1-2* and *adr1-L2 lsd1-2* both exhibited complete ion leakage suppression, while *adr1 lsd1-2* exhibited a marginal effect (Figure 1C). Thus, ADR1-L1 and ADR1-L2 are each required for *lsd1* rcd.

We noted that *adr1-L1* and *adr1-L2* exhibited non-allelic non-complementation (NANC), a rare genetic condition where plants that are heterozygous at both loci phenotypically resemble either homozygous single mutant. Thus, plants homozygous for *lsd1-2* and heterozygous for both *ADR1-L1* and *ADR1-L2* exhibited full suppression of *lsd1* rcd (Figure 1D). We also found that *adr1-L2* was fully recessive, whereas *adr1-L1* appeared to be semi-dominant (Figure 1D). NANC frequently indicates that the two genes act closely together or that the two proteins physically interact or are a part of the same protein complex, and that their overall dose is important for their shared function [33]. Because all three ADR1 proteins share significant amino acid identity, we speculated that lowering of the overall ADR1 dose might be sufficient to suppress *lsd1* rcd. Thus, the weak *adr1* rcd suppression phenotype might simply reflect low expression of *ADR1* relative to *ADR1-L1* and *ADR1-L2*. Quantitative RT-PCR analysis of gene specific mRNA levels confirmed that *ADR1* is expressed at lower levels than *ADR1-L1* and *ADR1-L2* under our growth conditions, consistent with this model (Figure 1E).

### ADR1-L2 is required at the specific site undergoing cell death

**Figure 2:**
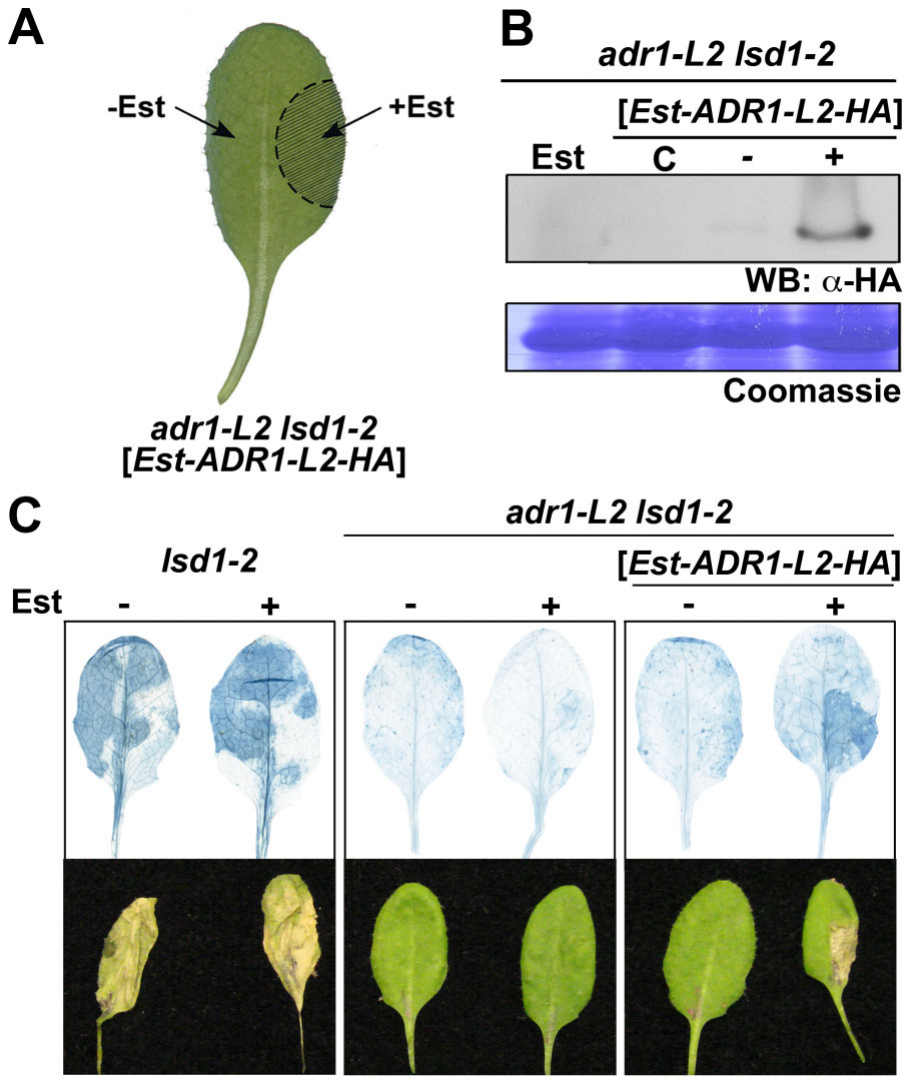
ADR1-L2 is required at the site undergoing cell death. (A) Schematic of the chimera. *adr1-L2 lsd1-2* expressing an estradiol inducible C-terminal HA-tagged ADR1-L2 were infiltrated in the indicated area with 20 µM estradiol, making that portion of the leaf *ADR1-L2 lsd1-2*. (B) Western blot to confirm expression of ADR1-L2 was limited to the estradiol-induced area. Estradiol + and − leaf areas were cored and protein was extracted from these cores. Protein extracts were run on SDS-Page gels and immunoblotted with anti-HA antibody. Coomassie stained blot confirms equal loading control (bottom). C, samples from un-infiltrated leaves; +, estradiol-infiltrated plant tissue; −, un-infiltrated tissue from the same leaf. In all samples, the entire leaf was treated with 300 µM BTH. (C) Trypan blue staining (top) of representative leaves (bottom) to show cell death in *lsd1* control and tissue chimera plants. Leaves from four-week-old plants were treated as indicated in (A). Plants were sprayed with BTH 16 hours after estradiol treatment, and leaves were stained with trypan blue 5 days after BTH treatment.

ADR1-L2 is a positive regulator of *lsd1*-mediated cell death. This could be due either to (i) a requirement for ADR1-L2 activation in cells destined to die, followed by its continued activation in neighboring cells, as the SA-dependent signal for rcd spreads in the absence of LSD1 [23], [34]; or (ii) a requirement for ADR1-L2 activation in cells initially triggered to die, with this activation contributing to the spread of an ADR1-L2-independent cell death signal beyond the primary cell death site. To distinguish between these two hypotheses, we generated an estradiol-driven (Est) conditional expression system, which induces local target gene expression [35]. *adr1-L2 lsd1-2* plants expressing an estradiol-induced, HA epitope-tagged *ADR1-L2* transgene were constructed (Materials and Methods). Expression of ADR1-L2 was activated by local application of estradiol on only part of a leaf, thus creating an artificial chimera containing both *adr1-L2 lsd1-2* and *ADR1-L2 lsd1-2* sectors (Figure 2A). ADR1-L2 expression was limited to the area of estradiol application as measured via Western blot (Figure 2B). BTH treatment was then used to induce *lsd1*-mediated rcd. We observed that cell death was limited to the zone of estradiol treatment and did not expand into the *adr1-L2 lsd1-2* sector (Figure 2C). This result supports our first hypothesis: ADR1-L2 expression is continuously required in cells undergoing *lsd1*-mediated rcd.

### The requirement for ADR1-L2 in *lsd1* rcd is P-loop dependent

**Figure 3:**
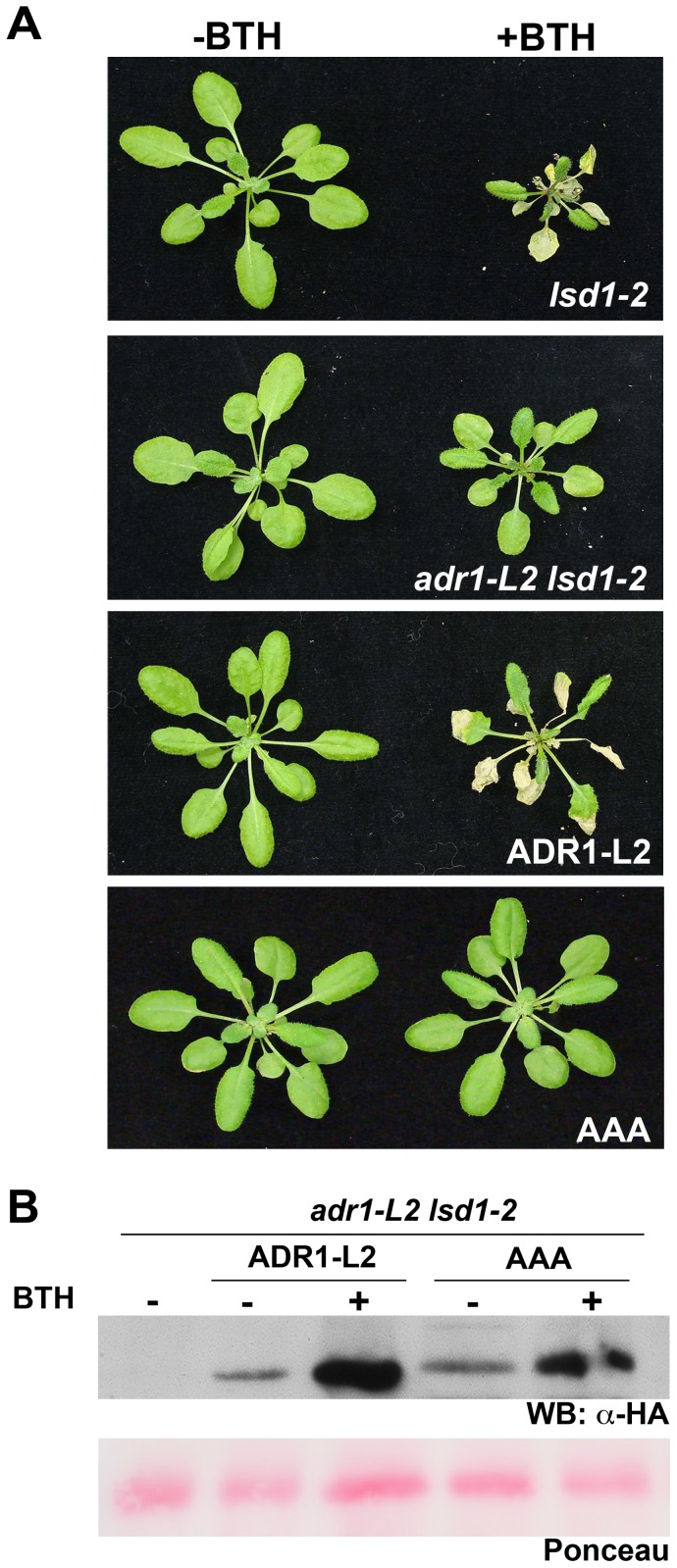
The requirement for ADR1-L2 in *lsd1* rcd is P-loop dependent. (A) Four-week-old plants of the indicated genotypes were sprayed with BTH or water. ADR1-L2 and AAA indicate *adr1-L2 lsd1* plants expressing C-terminally HA-tagged wild-type *ADR1-L2* or the mutated P-loop allele *ADR1-L2_AAA_*, respectively. In both transgenics expression is driven by the native *ADR1-L2* promoter. Pictures of plants were taken 5 dpi. (B) Protein from the indicated genotypes was extracted before or after BTH treatment, run on a denaturing gel, and probed with anti-HA antibody. Ponceau-stained blot shows relative loading.

We previously noted that ADR1-L2 is required for SA accumulation following effector and MAMP recognition, and that this does not require an intact P-loop motif [22]. However, these results do not preclude additional, canonical P-loop-dependent functions for ADR1-L2. Thus, we tested whether or not the positive regulatory function of ADR1-L2 in *lsd1* rcd is P-loop dependent. We generated *adr1-L2 lsd1-2* plants expressing ADR1-L2_AAA_, a mutated allele of ADR1-L2 which carries alanine (A) substitutions in the three consecutive conserved residues within the P-loop motif which are essential for nucleotide binding [22]. Interestingly, ADR1-L2_AAA_ fails to complement for *lsd1* rcd following BTH treatment (Figure 3A), even though this construct retains wild type BTH-induced ADR1-L2 protein accumulation (Figure 3B). Despite repeated attempts, we could not recover *adr1-L2* plants over-expressing ADR1-L2, presumably due to lethality of ectopic over-expression as noted for other sensor NB-LRR proteins (data not shown). Together these results suggest that ADR1-L2 activation in *lsd1* rcd proceeds in a canonical, P-loop dependent manner.

### An autoactive version of ADR1-L2 exhibits P-loop-dependent, ectopically activated immune responses

Mutations of the aspartic acid (D) in the conserved MHD motif in plant NB-LRRs typically lead to autoactivity [10]–[14]. Mechanistically, this is thought to reflect either a preference for ATP binding or a lack of ATPase activity, either of which would favor the “on” state, according to current models of NB-LRR activation [7], [19]. Thus, a similar mutation in the MHD motif of ADR1-L2 should result in a permanent ‘on’ state, resulting in ectopic autoactivity. In the cases where it has been examined, NB-LRR autoactivity via MHD mutation has been shown to require an intact P-loop [10]–[14]. Thus, given the P-loop dependent function of ADR1-L2 in *lsd1* rcd, we speculated that ADR1-L2 activity in additional defense contexts might also require an intact P-loop.

**Figure 4:**
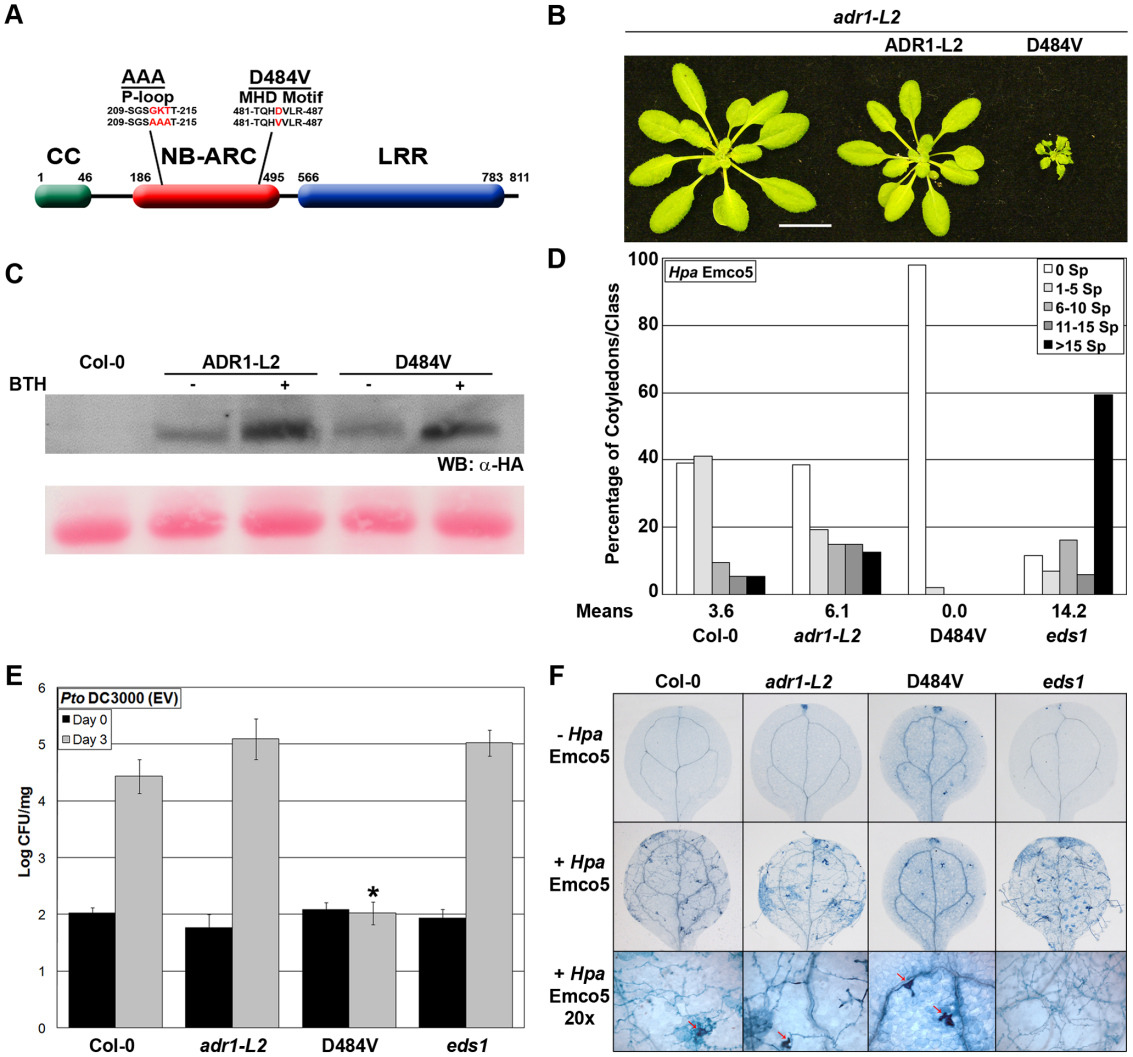
*ADR1-L2_D484V_* ectopically activates basal defense. (A) Schematic representation of ADR1-L2 showing the P-loop and MHD mutations used in this study. (B) Morphology of five-week-old *adr1-L2*, and *adr1-L2* complemented with *pADR1-L2::ADR1-L2-HA* or *pADR1-L2::ADR1-L2_D484V_-HA*, showing relative size. White bar is 2 cm. (C) Western blot of HA-tagged protein from the indicated genotypes before and after BTH application. Protein was extracted from plants, run on a denaturing gel and probed with anti-HA antibody. Ponceau-stained blot shows relative loading. (D) Ten-day-old seedlings were inoculated with 5×10^4^ sporangia/mL *Hpa* Emco5 via spray inoculation. Sporangiophores per cotyledon were counted 4 dpi, with an average of 80 cotyledons per genotype counted. Sporangiophore counts were classified into: no sporulation (0 sporangiophores/cotyledon), light sporulation (1–5), medium sporulation (6–10), heavy sporulation (11–15), or very heavy sporulation (>15). Means of sporangiophore per cotyledon are listed below the graph. (E) Twenty-day-old seedlings were dip-inoculated with *Pto* DC3000(EV). Bacterial growth was assayed at 0 and 3 dpi. Values are mean cfu/mg ±2× SE, n = 4. Asterisk indicates significant difference (Post Hoc test, p<0.0001). (F) Trypan blue stained leaves from (D) and magnified sites (20×). Leaves were collected and stained 4 dpi. Red arrows indicate HR sites.

We generated *adr1-L2* plants expressing *ADR1-L2* with a Val (V) for Asp (D) substitution at amino acid 484 (Figure 4A; hereafter *ADR1-L2_D484V_*). As expected, *ADR1-L2_D484V_* transgenics exhibited a dwarfed, *cpr* (*Constitutive PR expression*)-like phenotype [36] with short hypocotyls, pointed leaves (Figure 4B), and a bushy appearance after bolting. In contrast, *adr1-L2* plants expressing wild-type *ADR1-L2* appeared morphologically similar to wild-type Col-0 plants (Figure 4B). Both transgenes were expressed from the native *ADR1-L2* promoter, with C-terminal HA epitope tags (Figure 4C). We note that the majority of *ADR1-L2_D484V_* transgenic lines accumulated higher protein levels than those expressing the wild-type *ADR1-L2* allele. We selected *ADR1-L2* and *ADR1-L2_D484V_* lines expressing similar levels of protein to show that the *cpr*-like phenotype is not simply a result of higher protein levels in the autoactive mutant (Figure 4C); the differences in morphology persist. Additional *ADR1-L2_D484V_* lines expressing less ADR1-L2_D484V_ protein were also recovered; these did not exhibit strong *cpr*-like phenotypes, suggesting that there is a threshold amount of ADR1-L2_D484V_ required for the associated phenotypes (data not shown).

The *ADR1* family members work additively to limit pathogen growth, with *adr1* triple mutant plants exhibiting increased susceptibility to virulent pathogens [22]. We therefore tested the ability of autoactive ADR1-L2_D484V_ to confer enhanced basal defense against otherwise virulent pathogens. *ADR1-L2_D484V_* plants displayed increased resistance to both *Hyaloperonospora arabidopsidis* (*Hpa*) Emco5 and *Pseudomonas syringae* pv tomato (*Pto*) DC3000 (Figure 4D, 4E). Trypan blue staining of cotyledons after inoculation with *Hpa* Emco5 revealed predominantly free hyphal growth in the wild-type Col-0 control and *adr1-L2*, which was enhanced in the fully susceptible control, *eds1* (Figure 4F). *ADR1-L2_D484V_* plants, on the other hand, exhibited only localized hypersensitive cell death (HR) as well as a basal level of cell death (Figure 4F, top row) not seen in the other genotypes. Thus, ADR1-L2_D484V_ constitutively triggers downstream signaling and increased immune function.

**Figure 5:**
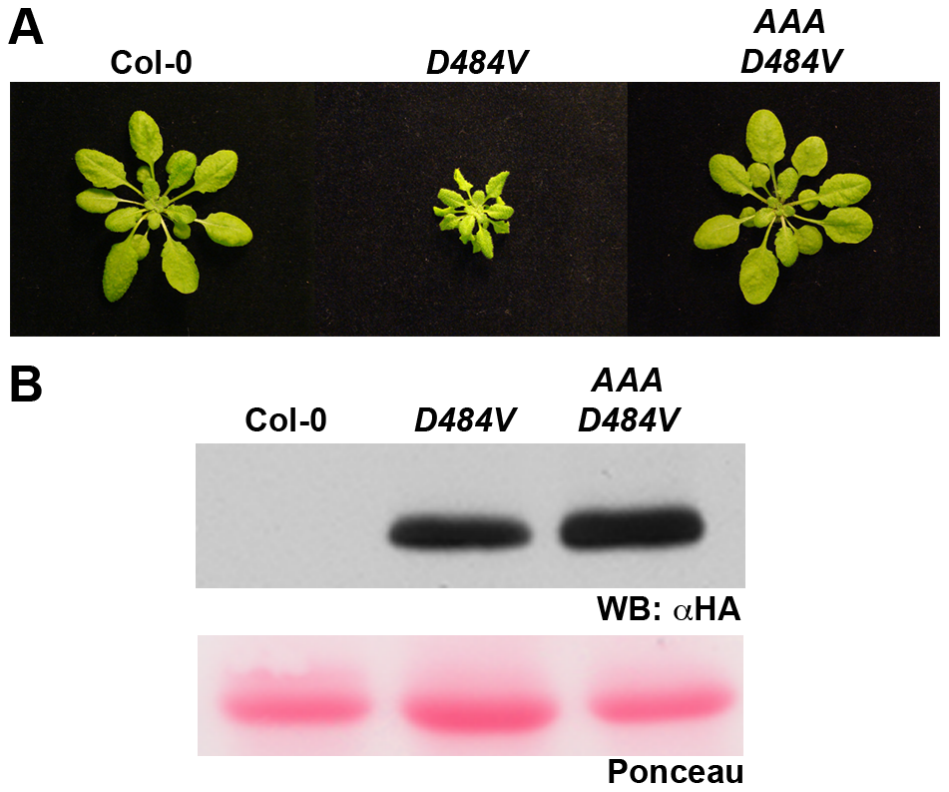
An intact P-loop catalytic domain is required for the *ADR1-L2_D484V_* morphological phenotype. (A) Pictures of five-week-old Col-0, *ADR1-L2_D484V_*, and *ADR1-L2_AAA D484V_* plants show relative morphology. (B) Western blot of Col-0 and HA-tagged ADR1-L2_D484V_ and ADR1-L2_AAA D484V_ protein from plants in (A). Relative loading indicated by Ponceau stained blot.

We examined the dependence of the *ADR1-L2_D484V_ cpr*-like phenotype on the P-loop. The triple missense P-loop dead mutation, *ADR1-L2_AAA_* [22], and the autoactive *ADR1-L2_D484V_* mutation were combined in *cis* (Figure 4A) and transformed into *adr1-L2* plants. *ADR1-L2_AAA D484V_* plants did not exhibit the *cpr*-like phenotype (Figure 5A) despite the fact that they expressed levels of ADR1-L2_AAA D484V_ protein that are similar to ADR1-L2_D484V_ levels sufficient to cause the dwarfed phenotype (Figure 5B). Thus, an intact P-loop domain is required for ADR1-L2_D484V_ autoactivity. We infer that ADR1-L2_D484V_ is an activated version of this NB-LRR which can be used to study the canonical, P-loop dependent functions of ADR1-L2.

### ADR1-L2_D484V_ autoactivity is regulated by *lsd1* suppressors

ADR1-L2 was identified as a positive regulator of *lsd1* rcd ([34], above). LSD1 and ADR1-L2 both function downstream of the NADPH oxidase-dependent ROI burst driven by NB-LRR sensor activation, but upstream of SA accumulation [22], [25], [26]. Additionally, ADR1-L2 is locally required for *lsd1*-mediated rcd and its function in this context is P-loop dependent (Figure 2, Figure 5). Thus, we hypothesized that genetic components known to regulate *lsd1* rcd might also be required for ADR1-L2_D484V_ activity. We generated double mutants between *ADR1-L2_D484V_* and the *lsd1* suppressors *sid2*, *eds1*, and *atmc1* to define genetic interactions required for the ADR1-L2_D484V_ phenotypes. We also generated *ADR1-L2_D484V_ atrbohD* double mutants to define whether an oxidative burst is required for the ADR1-L2_D484V_ phenotypes. We examined these double mutants for ADR1-L2_D484V_ protein accumulation, alterations in the *ADR1-L2_D484V_ cpr*-like morphology, enhanced resistance to the virulent *Hpa* isolate Emco5, and steady-state SA levels.

**Figure 6:**
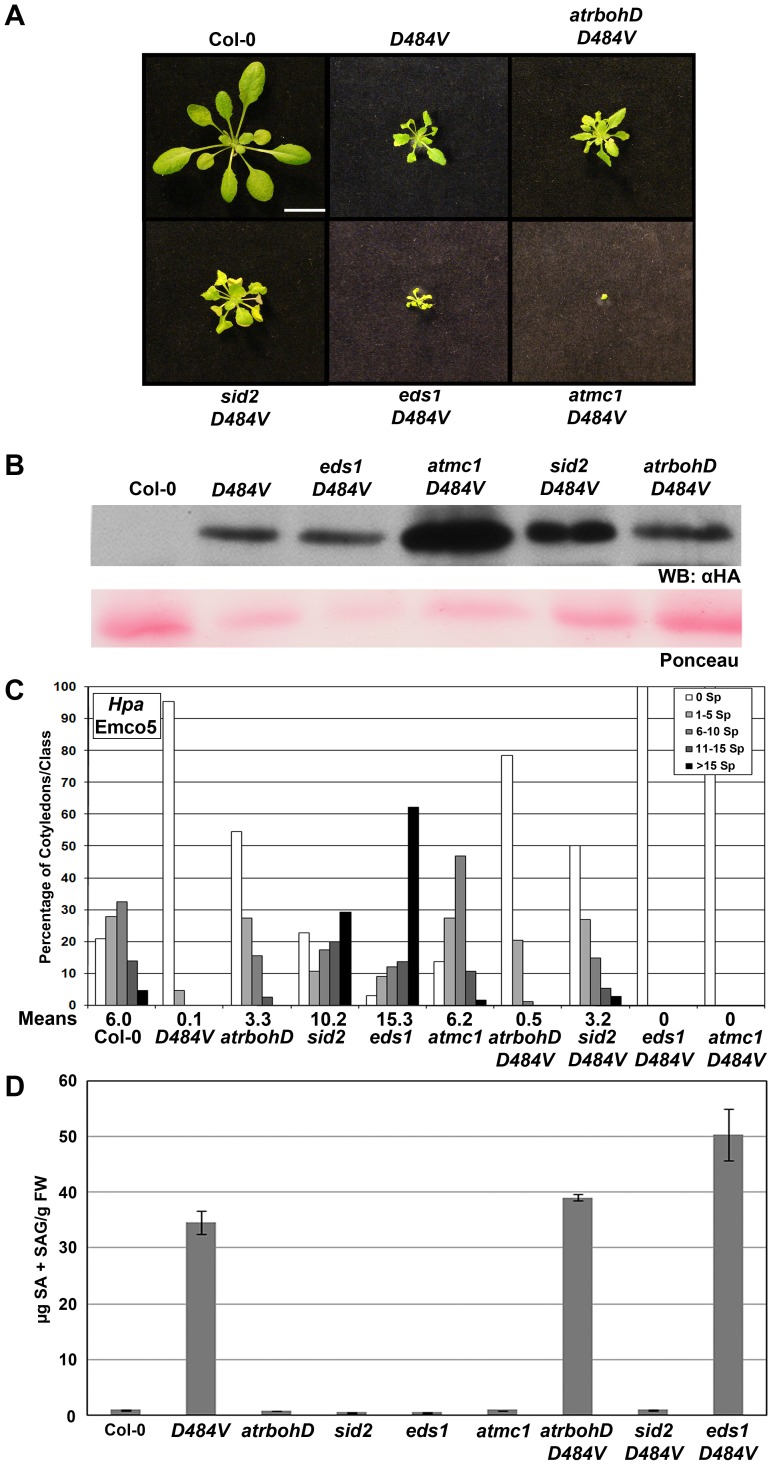
*lsd1* suppressors are regulators of *ADR1-L2_D484V_* autoactivity. (A) Pictures of five-week-old Col-0, *ADR1-L2_D484V_*, *atrbohD ADR1-L2_D484V_*, *sid2-1 ADR1-L2_D484V_*, *eds1-2 ADR1-L2_D484V_*, or *atmc1-1 ADR1-L2_D484V_* plants, showing morphological differences between the genotypes. White bar is 2 cm. (B) Western blots of HA-tagged ADR1-L2_D484V_ proteins from plants in (A). Ponceau staining shows relative loading. (C) Ten-day-old seedlings of the indicated genotypes were inoculated with 5×10^4^ sporangia/mL *Hpa* Emco5. At 4 dpi, sporangiophores were counted and classified as in Figure 4. Means per cotyledon are listed below the graph. (D) Steady-state total SA levels were measured from leaves of the indicated genotypes. Values are average µg of total SA from 4 replicates, ±2× SE.

*AtRbohD* is generally required for effector-driven, NB-LRR-dependent superoxide production, but not for *lsd1* rcd [23]. In fact, *lsd1-2 atrbohD* plants exhibit increased rcd compared to *lsd1-2* single mutants, a phenotype that depends on SA accumulation [25]. This result suggests that the NADPH oxidase can down-regulate the spread of cell death as SA-dependent signals emanate from an infection site [23]. *atrbohD ADR1-L2_D484V_* plants morphologically resembled the *ADR1-L2_D484V_* parent (Figure 6A, Figure S1) and expressed a similar level of ADR1-L2_D484V_ protein (Figure 6B). Like the *ADR1-L2_D484V_* parent, *atrbohD ADR1-L2_D484V_* plants were significantly more resistant to *Hpa* Emco5 (Figure 6C), and had extremely high steady-state levels of SA (Figure 6D). We conclude that ADR1-L2_D484V_ autoactivity, unlike effector-driven NB-LRR activation, is downstream, or independent, of AtRbohD.

SA is required for *lsd1* rcd [25] and mediates basal defense in plants [37]. Additionally, SA levels are reduced in *adr1*-family triple mutant plants, corresponding to diminished basal defense and an increase in disease susceptibility [22]. Thus, it seemed likely that the increased basal defense in *ADR1-L2_D484V_* plants could be due to the massive increase in SA observed in this line (Figure 6D). We tested this hypothesis using the *sid2* mutant, which is unable to synthesize SA due to a mutation in the biosynthetic isochorismate synthase gene, *ICS1* [16]. *sid2 ADR1-L2_D484V_* plants morphologically resembled the *ADR1-L2_D484V_* parent (Figure 6A, Figure S1) and accumulated similar amounts of ADR1-L2_D484V_ protein (Figure 6B). *sid2 ADR1-L2_D484V_* plants exhibited enhanced basal defense to *Hpa* Emco5, though not to the same extent as *ADR1-L2_D484V_* (Figure 6C). As expected, *sid2 ADR1-L2_D484V_* plants did not accumulate SA (Figure 6D). These observations indicate that the defense *cpr*-like phenotypes of *ADR1-L2_D484V_* consist of both SA-dependent and SA-independent components, whereas the *cpr*-like growth phenotype is SA-independent.

EDS1 is required for *lsd1*-mediated rcd [26] and is an essential regulator of both basal defense against virulent pathogens [38], [39] and TIR-NB-LRR dependent ETI [40]–[42]. Exogenous SA rescues *eds1* basal defense phenotypes, suggesting that EDS1 acts upstream of ICS1, at least for the phenotypes assayed [42], [43]. Importantly, *eds1 ADR1-L2_D484V_* plants were significantly more dwarfed than *ADR1-L2_D484V_* (Figure 6A, Figure S1), though these two lines expressed similar levels of ADR1-L2_D484V_ protein (Figure 6B). *eds1 ADR1-L2_D484V_* double mutants were completely resistant to *Hpa* Emco5 (Figure 6C), and had steady-state SA levels that were higher than the *ADR1-L2_D484V_* single mutant (Figure 6D). These surprising results demonstrate that EDS1 is a negative regulator of the SA-accumulation observed in ADR1-L2_D484V_.

AtMC1 is a metacaspase required for *lsd1* rcd; AtMC1 also contributes significantly to ETI-dependent HR [27]. *atmc1 ADR1-L2_D484V_* plants were extremely dwarfed (Figure 6A, Figure S1). However, these plants were not sterile; they produced small amounts of seed and had a very long life cycle compared to wild-type Col-0 or *ADR1-L2_D484V_* plants (data not shown). They also accumulated more ADR1-L2_D484V_ protein than the *ADR1-L2_D484V_* parent (Figure 6B). Cotyledons of the *atmc1 ADR1-L2_D484V_* plants were similar in size to those of *ADR1-L2_D484V_* plants, and we were thus able to perform *Hpa* infection assays. We determined that *atmc1 ADR1-L2_D484V_* cotyledons are completely resistant to *Hpa* Emco5 (Figure 6C). Due to the extremely small size of the *atmc1 ADR1-L2_D484V_* double mutant, we were unable to perform SA analysis on this line. Collectively, these data indicate that AtMC1 negatively regulates ADR1-L2_D484V_ protein accumulation, and likely subsequent SA accumulation leading to a hyper-*cpr* phenotype.

### *lsd1 ADR1-L2_D484V_* is lethal, and this lethality requires *EDS1*

ADR1-L2 is required for *lsd1*-mediated rcd [22]. We therefore examined whether ADR1-L2_D484V_ affects the *lsd1* phenotype. We crossed *lsd1-2* and *ADR1-L2_D484V_* plants, and in the F3 generation homozygous *ADR1-L2_D484V_* plants were selected via Basta resistance markers on the transgene (Materials and Methods). *ADR1-L2_D484V_* homozygotes were genotyped for *lsd1-2*; none were *lsd1-2* homozygous (Table S1). Additionally, we carried *lsd1-2* homozygous, *ADR1-L2_D484V_* heterozygous plants forward an additional generation, and again used the Basta resistance marker to identify homozygous *ADR1-L2_D484V_* plants. None were recovered. Next, we attempted to transform *lsd1-2* mutant plants with the same *ADR1-L2_D484V_* construct used in the *adr1-L2* transformation. No lines were recovered that expressed detectable levels of ADR1-L2_D484V_ protein, and no plants that were recovered displayed the dwarfed phenotype (data not shown). We concluded that *lsd1-2 ADR1-L2_D484V_* is lethal.

**Figure 7:**
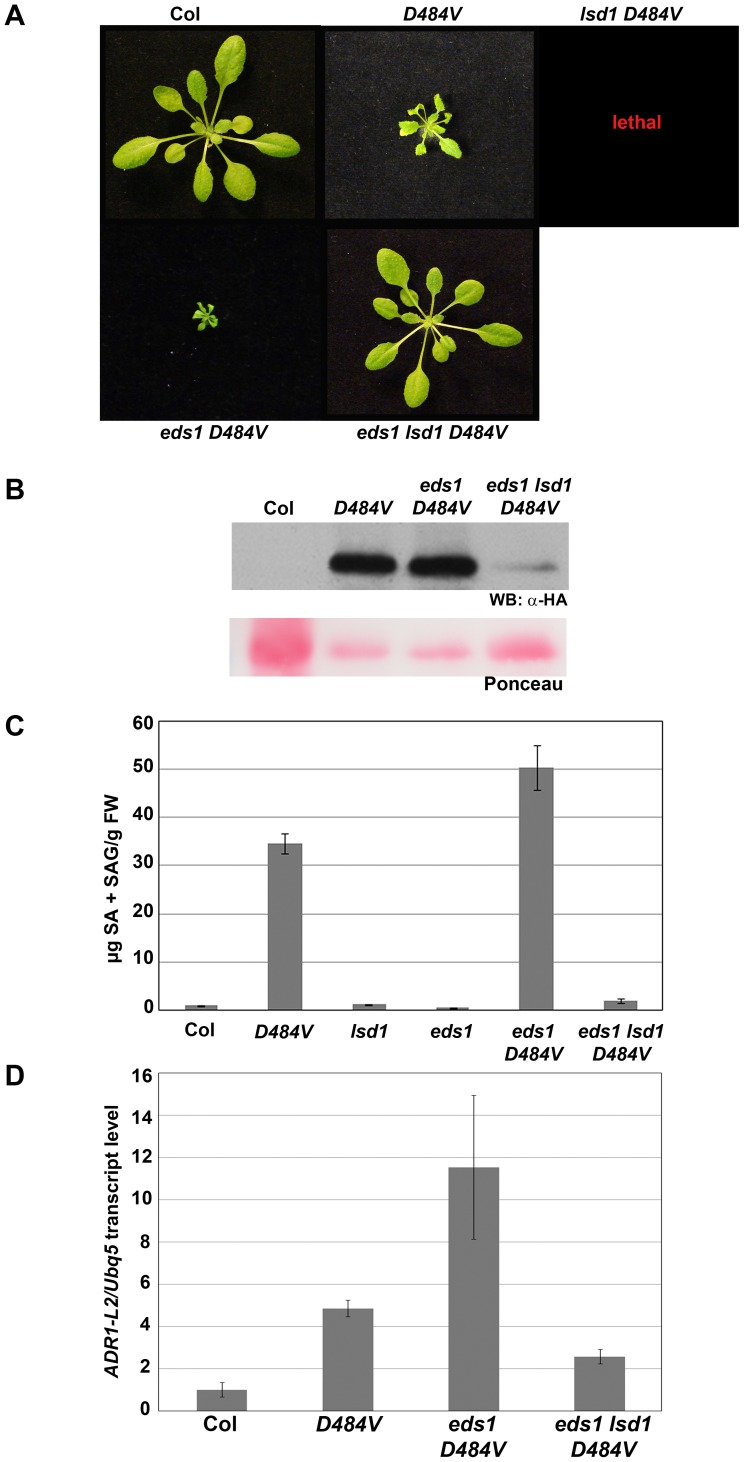
*eds1 lsd1 ADR1-L2_D484V_* plants lose ectopic activation phenotypes. (A) Pictures of five-week-old plants of the indicated genotypes show suppression of the *eds1-2 ADR1-L2_D484V_* phenotype in an *lsd1*-2 background. (B) Western blot of HA-tagged ADR1-L2_D484V_ protein from the indicated genotypes. Ponceau stain shows relative loading. (C) Total SA amounts (mean ±2× SE) were measured from plants of the indicated genotypes. Values are average µg of total SA from 4 replicates. Error bar represents ±2× SE. Controls here are from same experiment as data shown in Figure 6D. (D) Quantitative real time PCR for the transcript amounts of *ADR1-L2* in the indicated genotypes. Error bars represent ±2× SE.

We therefore looked for genetic determinants required for *lsd1 ADR1-L2_D484V_* lethality. As stated above, *eds1* and *atmc1* are both suppressors of *lsd1* rcd. We therefore crossed *atmc1 lsd1-2* or *eds1 lsd1-2* plants, which express wild-type growth, to *ADR1-L2_D484V_*. *atmc1 lsd1-2 ADR1-L2_D484V_* plants could not be recovered (data not shown), indicating that *AtMC1* is not required for lethality of *lsd1-2 ADR1-L2_D484V_*. However, we did recover *eds1 lsd1-2 ADR1-L2_D484V_* plants. These plants surprisingly exhibited wild-type morphology (Figure 7A), resembling *eds1 lsd1* [26]. The suppression of the *ADR1-L2_D484V_ cpr*-like phenotype is likely due to a much lower level of steady state ADR1-L2_D484V_ accumulation in the *eds1 lsd1-2 ADR1-L2_D484V_* plants compared to parental plants (Figure 7B). Despite examining many *eds1 lsd1-2 ADR1-L2_D484V_* plants from 4 independent progenies, no plant with *ADR1-L2_D484V_* parental expression levels was recovered. Additionally, *eds1 lsd1-2 ADR1-L2_D484V_* plants did not accumulate the high levels of SA observed in *ADR1-L2_D484V_* (Figure 7C).

In light of the surprising result that *eds1 lsd1-2 ADR1-L2_D484V_* plants are essentially wild-type, we re-confirmed the genotypes and phenotypes of *eds1 ADR1-L2_D484V_* and *eds1 lsd1-2 ADR1-L2_D484V_*. For this, we used a line that was homozygous for *eds1* and *ADR1-L2_D484_*_V_ but heterozygous for *LSD1* and expressed the wild-type morphology. In the next generation, both dwarfed and wild-type size plants were identified (Figure S2A). These plants were genotyped for *LSD1*, and all dwarfed plants were found to be *LSD1* homozygotes (Figure S2B, 20 of 70 plants were *LSD1* homozygotes). Wild-type size plants were either *LSD1/lsd1* heterozygotes (34 of 70 plants) or *lsd1* mutants (16 of 70 plants), suggesting that the dominant wild-type phenotype in this context is the result of *LSD1* haploinsufficiency. We therefore conclude that the difference in the phenotypes between *eds1 lsd1-2 ADR1-L2_D484V_* (wild-type) and both *eds1 ADR1-L2_D484V_* (nearly lethal) and *lsd1 ADR1-L2_D484V_* (lethal) is genuine. Further, in the presence of autoactive *ADR1-L2_D484V_*, the combined absence of EDS1 and the loss, or reduction, of LSD1 leads to down-regulation of ADR1-L2_D484V_ protein accumulation and restoration of wild-type morphology.

We addressed whether the lowered accumulation of ADR1-L2_D484V_ protein in *eds1 lsd1-2 ADR1-L2_D484V_* was due to transcriptional regulation. We performed quantitative RT-PCR, and discovered that the *ADR1-L2_D484V_* transcript levels in *eds1 lsd1-2 ADR1-L2_D484V_* plants were lower than in *ADR1-L2_D484V_* (Figure 7D), generally consistent with the diminution of ADR1-L2_D484V_ protein in *eds1 lsd1-2 ADR1-L2_D484V_* (Figure 7B). LSD1 and EDS1 are known to work together in an SA regulatory feedback loop [26]. Given that *eds1 lsd1-2 ADR1-L2_D484V_* plants are morphologically normal, express lower levels of SA than *ADR1-L2_D484V_*, and accumulate lower levels of *ADR1-L2* transcript and protein than *ADR1-L2_D484V_* (Figure 7), and that ADR1-L2 accumulation is up-regulated by BTH application (Figure 4C), we speculate that this loop also regulates *ADR1-L2* expression. In support of this hypothesis, we also noted that *ADR1-L2_D484V_* transcript accumulated to significantly higher levels than the endogenous *ADR1-L2* transcript in wild-type Col-0 plants (Figure 7D), indicating that plants expressing the activated ADR1-L2 allele constitutively up-regulate *ADR1-L2* transcription.

### ADR1-L2_D484V_ autoactivity is synergistically regulated by EDS1 function and SA accumulation

**Figure 8:**
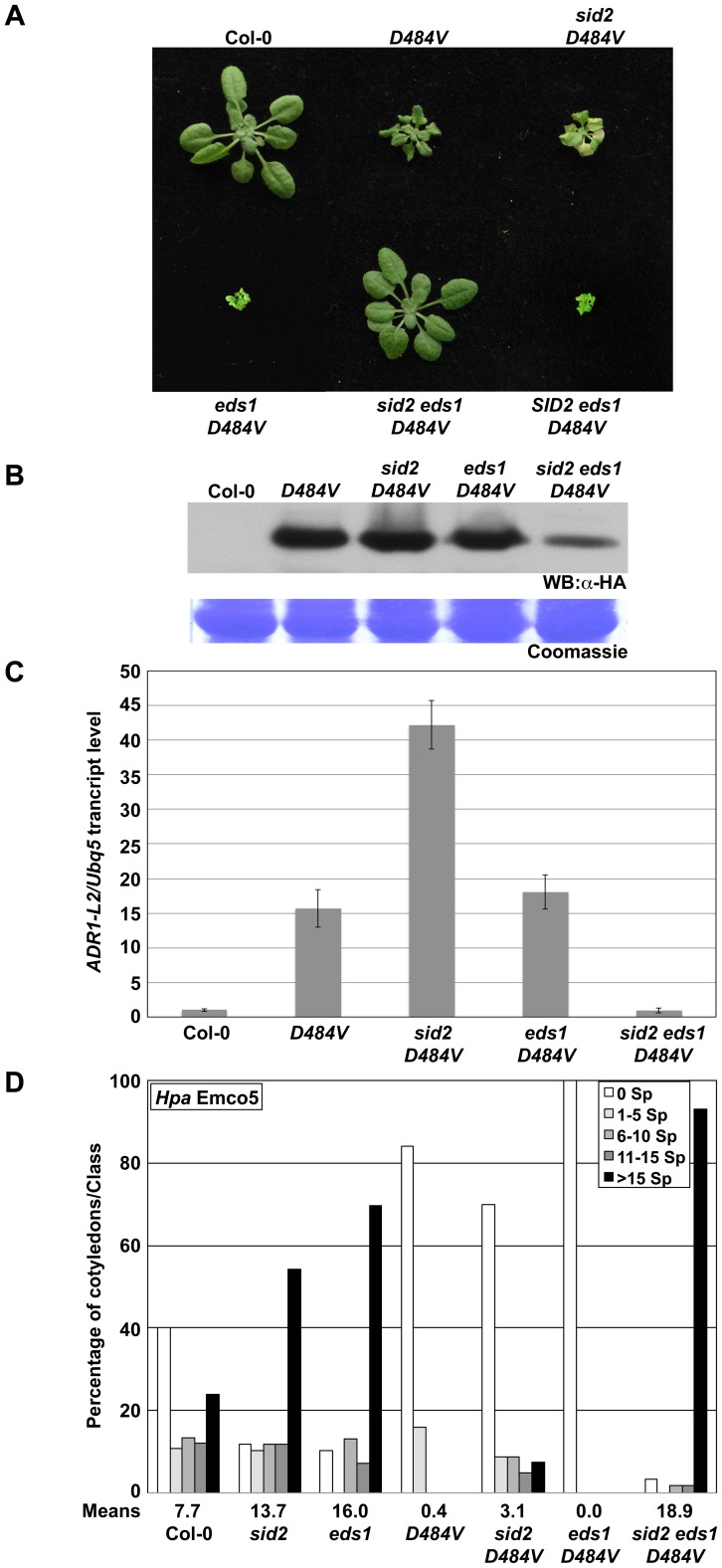
ADR1-L2_D484V_ autoactivity signaling requires both SA and EDS1. A) Pictures of five-week-old plants representative of the indicated genotypes. *SID2 eds1 ADR1-L2_D484V_* is a segregating F2 derived from the *eds1 ADR1-L2_D484V_*×*sid2 ADR1-L2_D484V_* cross. (B) Western blot of HA-tagged ADR1-L2_D484V_ protein from plants in (A). Coomassie stain shows relative loading. (C) Quantitative real-time PCR for the transcript amounts of *ADR1-L2* in the indicated genotypes. Error bar represents ±2×SE. (D) Ten-day-old seedlings were inoculated with 5×10^4^ sporangia/mL *Hpa* Emco5. At 4 dpi, sporangiophores were counted and classified as in Figure 4. Means per cotyledon are listed below the graph.

**Figure 9:**
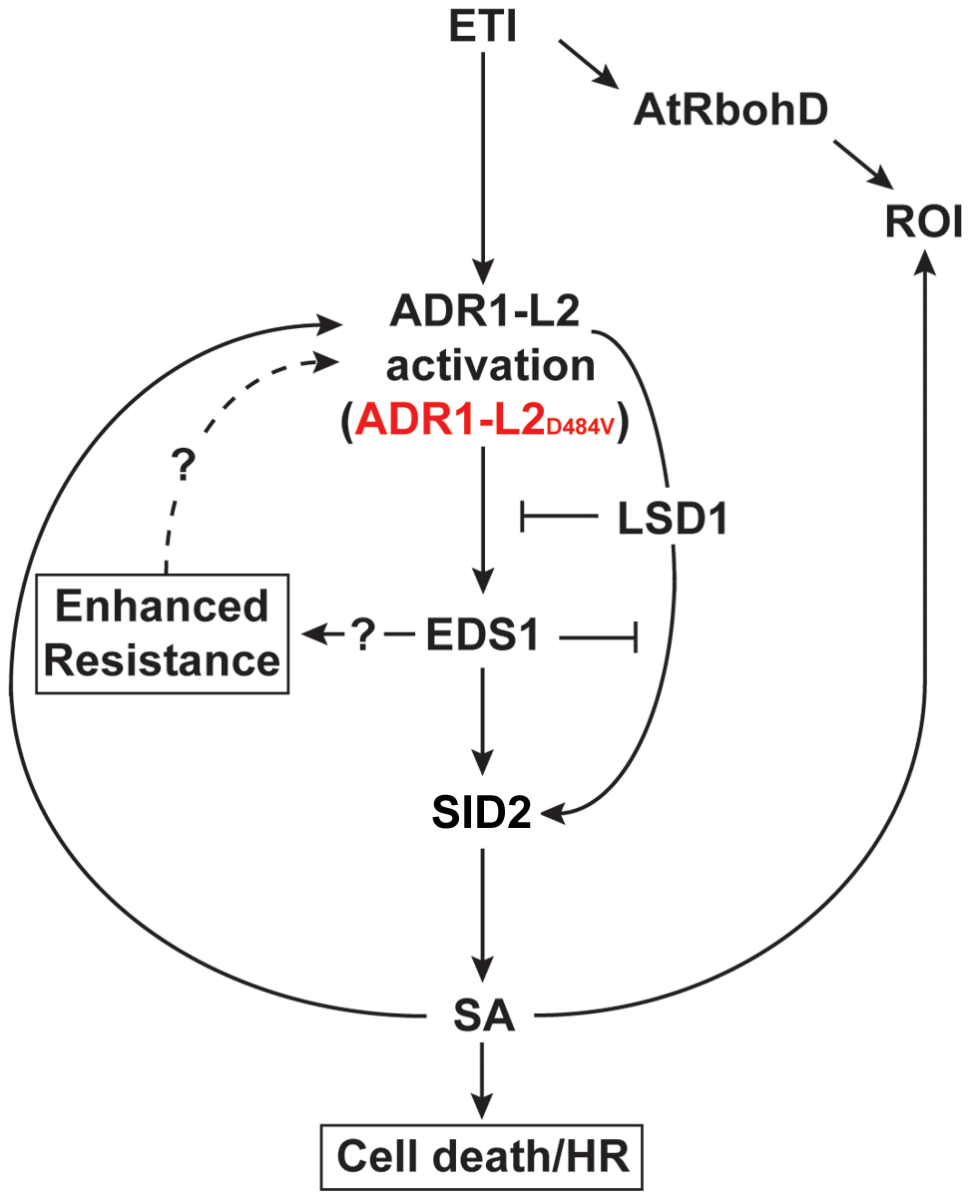
A model for the regulation of ADR1-L2_D484V_ activity. ETI activates both an AtRbohD-dependent ROI burst and SID2-dependent SA accumulation via ADR1-L2. Activated ADR1-L2 initiates cell death and disease resistance via SA-dependent and -independent pathways. EDS1 functions downstream of activated ADR1-L2 as a positive regulator of both SA accumulation and of the SA-independent pathway. ADR1-L2 also triggers SA via a pathway that is controlled by LSD1 and antagonized by EDS1. Therefore, the spread of this SA accumulation is spatially down-regulated through a combined action of EDS1 and LSD1. Due to its position in these feedback loops, SA functions both up- and down-stream of ADR1-L2.

The phenotypic suppression of *lsd1* lethality and of *eds1 ADR1-L2_D484V_* morphological defects in *eds1 lsd1 ADR1-L2_D484V_* plants suggests that ADR1-L2_D484V_ autoactivity signals via two parallel pathways leading to SA accumulation, one EDS1- and one LSD1-dependent. These converge through mutual negative regulation exerted by EDS1 on the LSD1-dependent pathway and vice versa. LSD1 dampens an SA regulatory feed-forward loop that requires EDS1 [26]. EDS1 dampens an LSD1-dependent SA-accumulation (Figure 7). Thus it is plausible that *eds1 lsd1 ADR1-L2_D484V_* resembles a wild-type plant because the SA levels cannot be feed-forward amplified. To test this hypothesis, we generated *sid2 eds1 ADR1-L2_D484V_* plants by crossing *sid2 ADR1-L2_D484V_* to *eds1 ADR1-L2_D484V_*. Similar to *eds1 lsd1 ADR1-L2_D484V_*, these plants exhibited complete suppression of the nearly lethal *eds1 ADR1-L2_D484V_* phenotype (Figure 8A). Additionally, the steady state accumulation of the transgene was lowered compared to either parental line (Figure 8B). We noted that the reduced protein accumulation was not caused by transgene silencing, as F2 progeny from *sid2 ADR1-L2_D484V_*×*eds1 ADR1-L2_D484V_* segregated the *SID2 eds1 ADR1-L2_D484V_* morphological phenotype (Figure 8A). Quantitative RT-PCR on *ADR1-L2* transcript suggested that, similar to *eds1 lsd1 ADR1-L2_D484V_*, the reduced transgene accumulation is transcriptional (Figure 8C). As noted above, an additional hallmark of *ADR1-L2_D484V_* autoactivity is enhanced immune function. We thus tested whether the enhanced basal defense response of *ADR1-L2_D484V_* is affected in the *eds1 sid2* mutant background. Strikingly, *sid2 eds1 ADR1-L2_D484V_* plants were extremely susceptible to *Hpa* Emco5, more so than either single *sid2* or *eds1* mutants (Figure 8D). A model consistent with these observations and previous publications is presented in Figure 9 and discussed below.

### RAR1 is dispensable for accumulation of ADR1-L2

The autoactive phenotypes of *ADR1-L2_D484V_* plants require ADR1-L2_D484V_ protein accumulation above a threshold. This indicates that the expression level of wild-type *ADR1-L2* may also be under exquisite control. The co-chaperone RAR1, while not necessary for the function of all NB-LRRs, is required for the steady state accumulation of all NB-LRRs tested to date [44]–[47]. We thus crossed *adr1-L2 pADR1-L2:ADR1-L2-HA* to *rar1-21* [46]. Plants genotyped as homozygous *rar1-21* and homozygous *RAR1* exhibited similar levels of ADR1-L2-HA protein (Figure S3A), indicating that RAR1 is not required for ADR1-L2 accumulation. The *rar1* genotype was confirmed by Western blot for RAR1 protein (Figure S3B). ADR1-L2 expression can be up-regulated with BTH [22]. We therefore also tested whether RAR1 is required for the high levels of ADR1-L2 accumulating after BTH treatment. BTH induced ADR1-L2 protein in *rar-21 ADR1-L2-HA* plants accumulated to levels at least as high as those in *RAR1 ADR1-L2-HA* plants (Figure S3A). Therefore, RAR1 is dispensable for both steady-state ADR1-L2 accumulation, in contrast to other assayed NB-LRR proteins [44]–[47], and for its BTH-induced up-regulation.

## Discussion

We recently demonstrated that the plant NB-LRR immune receptor ADR1-L2 can have non-canonical, P-loop independent ‘helper’ functions in plant defense [22]. Here, we sought first to define canonical, P-loop dependent function(s) for ADR1-L2, and then to understand the genetic requirements for these functions. We demonstrated that wild-type ADR1-L2 is required locally at the site of BTH-driven cell death activation in the *lsd1* cell death control mutant. This activity requires an intact P-loop and is thus canonical. In this context, *ADR1-L2* genetically interacts with *ADR1-L1* to control runaway cell death, as shown by NANC, further suggesting that members of the ADR1 family function together in cell death signaling. ADR1-L2 does not require RAR1 for either its steady state accumulation, nor for its induced accumulation following BTH treatment. This is the first report of either steady state or inducible NB-LRR accumulation that is not RAR1-dependent. This result may differentiate ‘helper’ NB-LRRs from ‘sensor’ NB-LRRs. We propose that levels of the former might be dictated by the signaling partners with which they function in specific stoichiometries, while the latter, acting as effector-sensors, are threshold-regulated by the NB-LRR co-chaperone complex [48].

Given the canonical P-loop-dependent function of ADR1-L2 as a positive regulator of *lsd1* cell death, we inferred that ADR1-L2, like other NB-LRRs studied to date, retains the ability to undergo a nucleotide-dependent conformational switch to regulate its activation. Thus, we sought a context in which we could analyze canonical ADR1-L2 P-loop dependent functions, despite the absence of an effector to trigger it. We created an autoactive allele, *ADR1-L2_D484V_*. *ADR1-L2_D484V_* plants exhibit the dwarfed morphology and constitutively active defense responses observed in other autoactive NB-LRR mutants. We showed that this autoactivity requires an intact P-loop. We then used this allele as a proxy for canonical activation of ADR1-L2 in a series of epistasis experiments. We present a model consistent with our new findings and previous genetic analyses [22], [23], [25], [26], [30] (Figure 9).

Canonical, P-loop dependent, ‘sensor’ NB-LRR functions typically drive both the AtrbohD NADPH oxidase-dependent oxidative burst following effector perception and SID2-dependent SA accumulation [23]. By contrast, ADR1-L2_D484V_ autoactivity is downstream, or independent, of AtrbohD, yet still drives SID2-dependent SA accumulation. This is consistent with the previously defined, P-loop-independent ‘helper’ activity of ADR1-L2 [22].

Plants expressing *ADR1-L2_D484V_* exhibited increased disease resistance and very high steady-state levels of SA. *sid2 ADR1-L2_D484V_* plants expressed, as expected, very low levels of SA, but these plants did not completely revert to wild-type morphology, and they maintained an increased level of enhanced disease resistance. Thus, there must be SA-independent regulation of activated ADR1-L2. Redundant functions of EDS1 and SA in plant defense mediated by ‘sensor’ NB-LRR functions have been reported [30]. In that work, *sid2* or *eds1* mutants were insufficient to disrupt CC-NB-LRR-mediated disease resistance, while combined loss of both gene products led to loss of resistance [30]. Our results support this model, since the constitutive activation of ADR1-L2_D484V_ results in both SA-dependent and SA-independent phenotypes (Figure 9). Given these data, as well as the fact that *eds1 lsd1 ADR1-L2_D484V_* phenocopies *sid2 eds1 ADR1-L2_D484V_* we conclude that the SA-independent pathway we describe here requires EDS1 (Figure 9, left).

One of our most surprising observations is the phenotypic rescue of both the lethal *lsd1 ADR1-L2_D484V_* phenotype and the nearly lethal *eds1 ADR1-L2_D484V_* phenotype in *eds1 lsd1 ADR1-L2_D484V_* plants. It is important to recall that either *adr1-L2* or *eds1* suppresses *lsd1* rcd [22], [26]. Recall also that the P-loop independent function of ADR1-L2 as a ‘helper’ is downstream of AtRbohD, but upstream of SA accumulation [22]. This is in agreement with the autoactive *ADR1-L2_D484V_* phenotype, which bypasses AtRbohD but still drives enhanced SA levels. Notably, loss of LSD1 in the *eds1 ADR1-L2_D484V_* context functionally resembles loss of SID2. Since SID2-dependent SA accumulation is regulated by LSD1, we conclude that both SA and EDS1 are required for *ADR1-L2_D484V_* autoactivity. Loss of either genetic component destroys the fine-tuned equilibrium between EDS1-dependent and SA-dependent processes in this autoactivity.

P-loop-dependent activation of ADR1-L2 results in SID2-dependent SA accumulation via two separate pathways (Figure 9). In the first pathway, ADR1-L2_D484V_ constitutively signals to EDS1, which in turn positively regulates SID2, increasing SA levels. ADR1-L2_D484V_ also triggers additional SA production in a parallel pathway that requires LSD1 and is antagonized by EDS1. In support of our model, SA regulates EDS1 transcription [29], which in turn regulates SID2 [49]. Once activated, ADR1-L2 causes cell death, which drives more AtRbohD-dependent ROI [34] and SA accumulation in surrounding cells [34], [50]. In both pathways, SA is part of a feedback loop that further potentiates the P-loop dependent activity of ADR1-L2, as indicated by the fact that ADR1-L2 is BTH inducible. Thus, ADR1-L2 is also both upstream and downstream of SA accumulation (Figure 9).

Our data are consistent with ADR1-L2 transcriptional regulation by both SA-dependent and -independent pathways (Figure 9). In an otherwise wild-type plant expressing activated ADR1-L2, the antagonism between EDS1 and LSD1 maintains SA production below toxic levels. In an *lsd1* plant, the level of SA surpasses this level via ectopic ADR1-L2 activation and consequent SA production. This increased SA in turn drives higher ADR1-L2 expression, and the cycle repeats. This is exacerbated, and lethal, in *lsd1 ADR1-L2_D484V_*. *eds1* and *sid2* suppress *lsd1* because feed forward regulation of the SA accumulation cycle is blocked. The surprising *eds1 lsd1 ADR1-L2_D484V_* and *sid2 eds1 ADR1-L2_D484V_* phenotypes are consistent with the low level of SA in these lines being insufficient to up-regulate *ADR1-L2* expression. Thus, even though there is chronic signaling feeding the cycle in *ADR1-L2_D484V_*, the EDS1-dependent, SA-independent pathway is interrupted in *eds1 lsd1 ADR1-L2_D484V_* and *sid2 eds1 ADR1-L2_D484V_*. How LSD1 and EDS1 negatively regulate each other has yet to be determined, although our data suggest that LSD1 might regulate EDS1 function through transcriptional control, as *EDS1* transcription levels are increased in an *lsd1* mutant (Figure S4). In support of this hypothesis, a role for LSD1 as a cytosolic retention factor for the AtbZIP10 transcription factor [51] may provide a mechanism for LSD1 control of *EDS1* expression.

Our model (Figure 9) supports a scenario in which in wild-type, P-loop dependent NB-LRR activation leads to local increased levels of SA via an AtRbohD-dependent ROI burst and SID2-dependent SA accumulation. The spread of this SA accumulation is spatially down-regulated through a combined action of EDS1 and LSD1 at increasing distance from the infection site. As stated above, our model also implies that SA functions both up- and down-stream of ADR1-L2. This may readily reconciled with our previous finding that ADR1-L2 helper function is required for SA accumulation and cell death, since ARD1-L2 is SA-up-regulated [22].

Overall, we present a general approach to characterize canonical, P-loop dependent functions of NB-LRR proteins in the absence of a specific effector. We applied this to a recently characterized ‘helper’ NB-LRR protein, ADR1-L2. We identified genetic components that regulate its P-loop-dependent, canonical functions, and found that they, in turn, are regulated by suppressors of the *lsd1* rcd phenotype. Our work suggests that the genetic requirements for ‘helper’ NB-LRR function may differ from the effector-driven activation of canonical ‘sensor’ NB-LRRs. Given that ADR1-L2, unlike other NB-LRRs, is required for *lsd1* rcd, we note that our results may be mainly relevant to the dissection of the functions of ADR1-L2 and its paralogues, rather than being broadly applicable to understanding of ‘sensor’ NB-LRRs. Nevertheless, in agreement with previous reports on ‘sensor’ NB-LRR function [30], we conclude that the P-loop-dependent autoactivity of ADR1-L2 relies on signaling pathways that differ in their requirement for SA accumulation, but which are both regulated by EDS1. Thus, though the requirements for ‘sensor’ and ‘helper’ NB-LRR functions may be separable, they could still share some overlapping features.

A significant challenge remains to address the sub-cellular localization of these regulatory circuits [52]. Resting state NB-LRRs are localized to diverse sub-cellular compartments, and dynamic re-localization may accompany effector-driven activation of some [19]. Defining any dynamics of protein localization associated with the differential ADR1-L2 canonical and non-canonical functions will be ultimately important for understanding the genetic network that we describe.

## Materials and Methods

### Plant lines and pathogen strains

All *Arabidopsis* lines are in the Columbia (Col-0) ecotype. *adr1-1* [22], *adr1-L1-1* [22], *adr1-L2-4* [22], *eds1-2* [49], *sid2-1*, *atrbohD* [23], *lsd1-2* [24], *atmc1* [27], and *rar1-21* [46] are described elsewhere; primers used to genotype these lines are in Table S2. For generation of *adr1-L2* plants expressing *ADR1-L2-HA*, ADR1-*L2_D484V_-HA*, and *ADR1-L2_AAA D484V_-HA* lines, the C-terminal HA-tagged coding sequence of wild-type *ADR1-L2* or the mutated alleles were fused to its native promoter (500 bp) and cloned in the pBAR (Basta resistant) Gateway vector [53]. For generation of *adr1-L2 lsd1-2* plants expressing an estradiol inducible ADR1-L2-HA, the coding sequence of ADR1-L2 was cloned into a modified pMDC7 (hygromicin resistant) Gateway vector carrying a C-terminal HA tag. Arabidopsis transgenics were generated using Agrobacterium (GV3101)-mediated floral dip transformation [54]. Basta selection of transgenic plants was performed by spraying 10-day-old seedlings. Plants were grown under short day conditions (9 hrs light, 21°C; 15 hrs dark, 18°C).

### Immunoblot analysis

Leaves from 4-week-old plants were harvested and total proteins were extracted by grinding frozen tissue in a buffer containing 20 mM Tris-HCl (pH 7.0), 150 mM NaCl, 1 mM EDTA (pH 8.0), 1% Triton X-100, 0.1% SDS, 10 mM DTT, and plant protein protease inhibitor cocktail (Sigma-Aldrich). Samples were centrifuged at 14,000 rpm for 15 min at 4°C to pellet debris. Proteins were separated on 7.5% (ADR1-HA) or 12% (RAR1) SDS-PAGE gels and were transferred to polyvinylidene difluoride membrane. Western blots were performed using standard methods. Anti-HA (Santa Cruz Biotechnology) antibody was used at a 1∶3000 dilution; anti-RAR1 (custom anti-RAR1 polyclonal antibody was made against the full length RAR1 with C-terminus GST tag by Cocalico Biologicals, Inc.) was used at a 1∶2000 dilution. Signals were detected by enhanced chemiluminescence using ECL Plus (Amersham Biosciences). For BTH induction experiments (300 µM), plants were collected 24 hpi.

### SA measurement

SA and SAG measurements were performed as described [55]. Briefly, 100 mg of leaves were collected from 4-week-old plants and frozen in liquid nitrogen. Samples were ground and tissue was homogenized in 200 µl 0.1M acetate buffer pH 5.6. Samples were centrifuged for 15 min at 16,000 g at 4°C. 100 µl of supernatant was transferred to a new tube for free SA measurement, and 10 µl was incubated with 1 µl 0.5 U/µl β-glucosidase for 90 min at 37°C for total SA measurement. After incubation, plant extracts were diluted 5-fold with 44 µl acetate buffer for free SA measurement. 60 µl of LB, 5 µl of plant extract (treated or not with β-glucosidase), and 50 µl of *Acinetobacter sp.* ADPWH-lux (OD = 0.4) were added to each well of a black 96-well plate (BD Falcon). The plate was incubated at 37°C for 60 min and luminescence was read with Spectra Max L (Molecular Devices) microplate reader. For the standard curve, 1 µl of a known amount of SA (Sigma; from 0 to 1000 µg/ml) was diluted 10-fold in *sid2-1* plant extract, and 5 µl of each standard (undiluted for free SA measurement, or 5-fold diluted for total SA) was added to the wells of the plate containing 60 µl of LB and 50 µl of *Acinetobacter*. SA standards were read in parallel with the experimental samples. For BTH induction experiments (300 µM), plants were collected 24 hpi.

### Pathogen strains and growth quantification

Ten-day-old seedlings were spray-inoculated with 50,000 spores/ml of *Hyaloperonospora arabidopsidis* isolate Emco5. Pots were covered with a lid to increase humidity during inoculation and pathogen growth. Sporangiophores were counted at 4 dpi as described [56]. *Pto* DC3000(EV) was resuspended in 10 mM MgCl_2_ to a final concentration of 2.5×10^5^ cfu/ml (OD_600_ = 0.0005). Twenty-day-old seedlings were dipped in the bacterial solution and growth was assessed as described [57].

### Cell death assays

4-week-old plants were sprayed with 300 µM BTH, or 10-day-old plants were inoculated with *Hpa* Emco5 as described above. Leaves were harvested and stained with lactophenol Trypan Blue (TB) to visualize dead cells as described [58]. For the conductivity measurements, 4-week-old plants were sprayed with 300 µM BTH. Plants were harvested and 4 leaf discs (7 mm) were cored and then floated in water for 30 min. These leaf discs were transferred to tubes containing 6 ml distilled water. Conductivity of the solution (μSiemens/cm) was determined with an Orion Conductivity Meter at the indicated time points [59].

### Creation of an artificial chimera

The central portion of the right halves of leaves from 4-week-old transgenic *adr1-L2 lsd1-2* plants expressing an estradiol inducible allele of ADR1-L2 were hand-infiltrated with Est (20 µM) using a needleless syringe. 300 µM BTH was sprayed on the whole plant 24 h post-Est application. 20 µM Est was then hand-infiltrated on the same portion of the leaves 2 dpi to ensure expression of ADR1-L2. Leaves were collected 5 dpi from the first Est infiltration.

### Quantitative RT–PCR

Leaves from 4-week-old plants were collected, frozen into liquid nitrogen and ground into powder with a mortar and pestle. RNA was extracted using TRIzol (Invitrogen), DNased (Ambion Turbo DNase), and cleaned up with Qiagen RNeasy Mini kit. Reverse transcription was performed (Ambion RETROscript) using 1 µg total RNA, and cDNA was analyzed with SYBR green (Applied Biosystem) using an Applied Biosystems ViiA7. Primers used are listed in Table S2.

### Selection of segregating plants

Pots of sibling plants fixed for *eds1* and segregating *lsd1-2* (*LSD1* heterzygotes) were Basta sprayed to check for segregation of *ADR1-L2_D484V_*. Those found to be *eds1 ADR1-L2_D484V_* were transplanted individually into pots, monitored for size, and genotyped for the T-DNA insertion of the *lsd1-2* mutation.

## Supporting Information

Figure S1 Quantification of plant growth based on fresh weight measurement. Five-week-old rosettes of the indicated genotypes were weighed. Means are representative of 10 plants for each genotype. Error bars indicate ±2× SE.
(TIF)

Figure S2 *eds1 ADR1-L2_D484V_* plants segregating *LSD1* show both wild-type and extreme *cpr* phenotypes. (A) Pictures of plants homozygous for *eds1* and *ADR1-L2_D484V_* and segregating *lsd1*. (B) PCR genotyping of the indicated genotypes confirms that only *LSD1* homozygous *eds1 ADR1-L2_D484V_* (#5) plants have the severely stunted growth phenotype. #1 and 2 indicate the Col-0 and *lsd1-2* controls respectively, #3–5 represent the genotypes from (A).
(TIF)

Figure S3 RAR1 is not required for either steady state ADR1-L2 accumulation or BTH-mediated induction. (A) *ADR1-L2-HA* and *rar1-21 ADR1-L2-HA* plants were sprayed with 300 µM BTH. Plants were collected for protein extraction 24 hpi. Protein from Col-0, *rar1-21*, and *ADR1-L2-HA* and *rar1-21 ADR1-L2-HA* plants + and -BTH were run on denaturing gels and probed with anti-HA antibody. (B) Protein from plants in (A) was also used in an anti-RAR1 Western blot to confirm the *rar1-21* genotype. Ponceau stained blots in (A) and (B) show relative loading.
(TIF)

Figure S4 LSD1 negatively regulates *EDS1* transcript. Quantitative real time PCR for the transcript amounts of *EDS1* in Col-0, *eds1-2*, and *lsd1-2*.
(TIF)

Table S1 *ADR1-L2_D484V_* is lethal in an *lsd1-2* background. Table of actual and expected genotypes of F3 progeny from a cross between *lsd1-2* and *ADR1-L2_D484V_* shows that no *lsd1-2* homozygous plants were recovered from plants that were homozygous for *ADR1-L2_D484V_*. *ADR1-L2_D484V_* was also transformed into *lsd1-2*, but no plants with a detectable amount of ADR1-L2_D484V_ protein were recovered.
(DOCX)

Table S2 Primer sequences used in this work.
(DOCX)
