# Supplementary figures and images for: Genetic Requirements for Signaling from an Autoactive Plant NB-LRR Intracellular Innate Immune Receptor
Source: PLoS Genet. 2013 Apr 25;9(4):e1003465. doi: 10.1371/journal.pgen.1003465 (PMC3636237; doi:10.1371/journal.pgen.1003465)

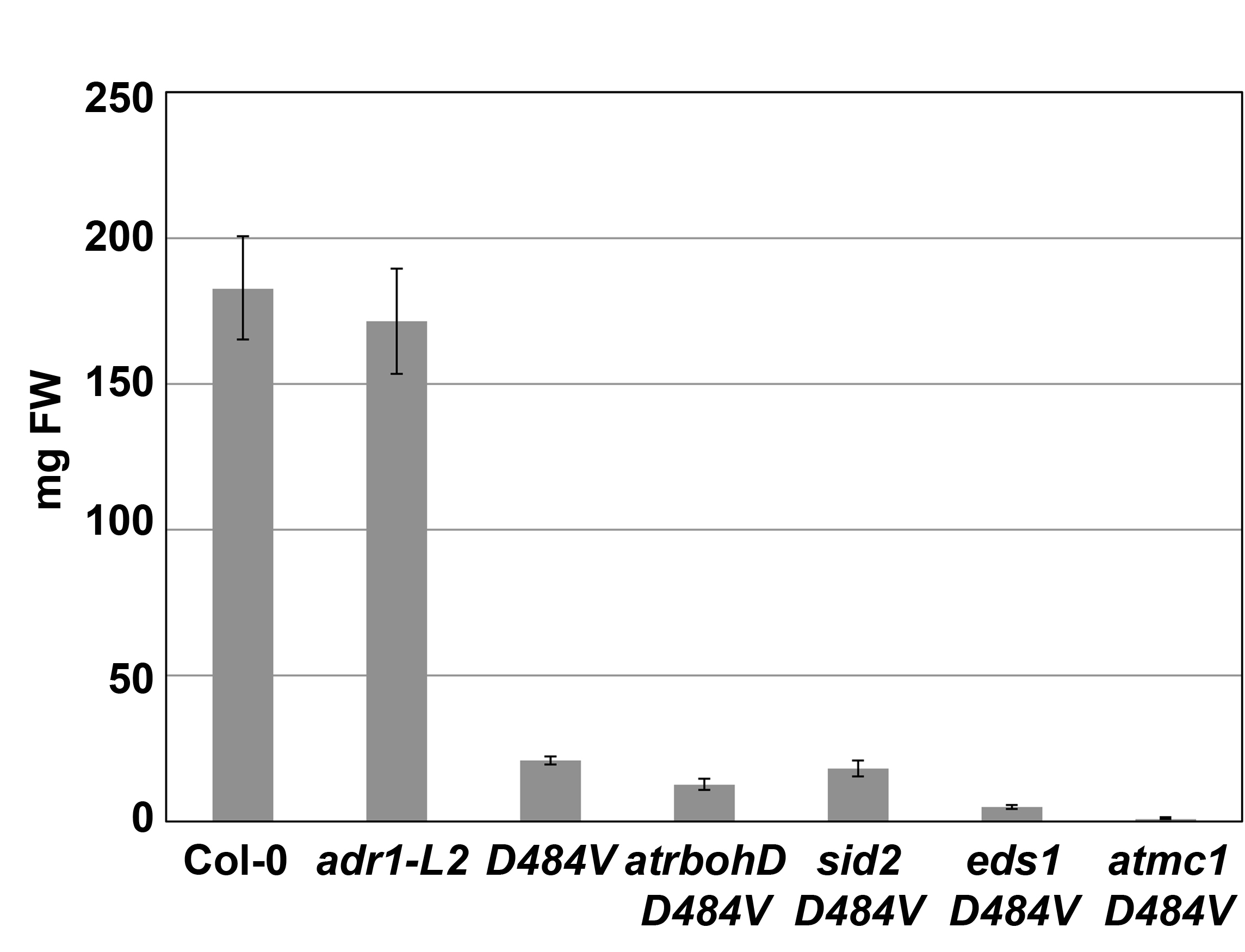

Supplement: Figure S1 — Quantification of plant growth based on fresh weight measurement. Five-week-old rosettes of the indicated genotypes were weighed. Means are representative of 10 plants for each genotype. Error bars indicate ±2× SE. (TIF) [file pgen.1003465.s001.tif]

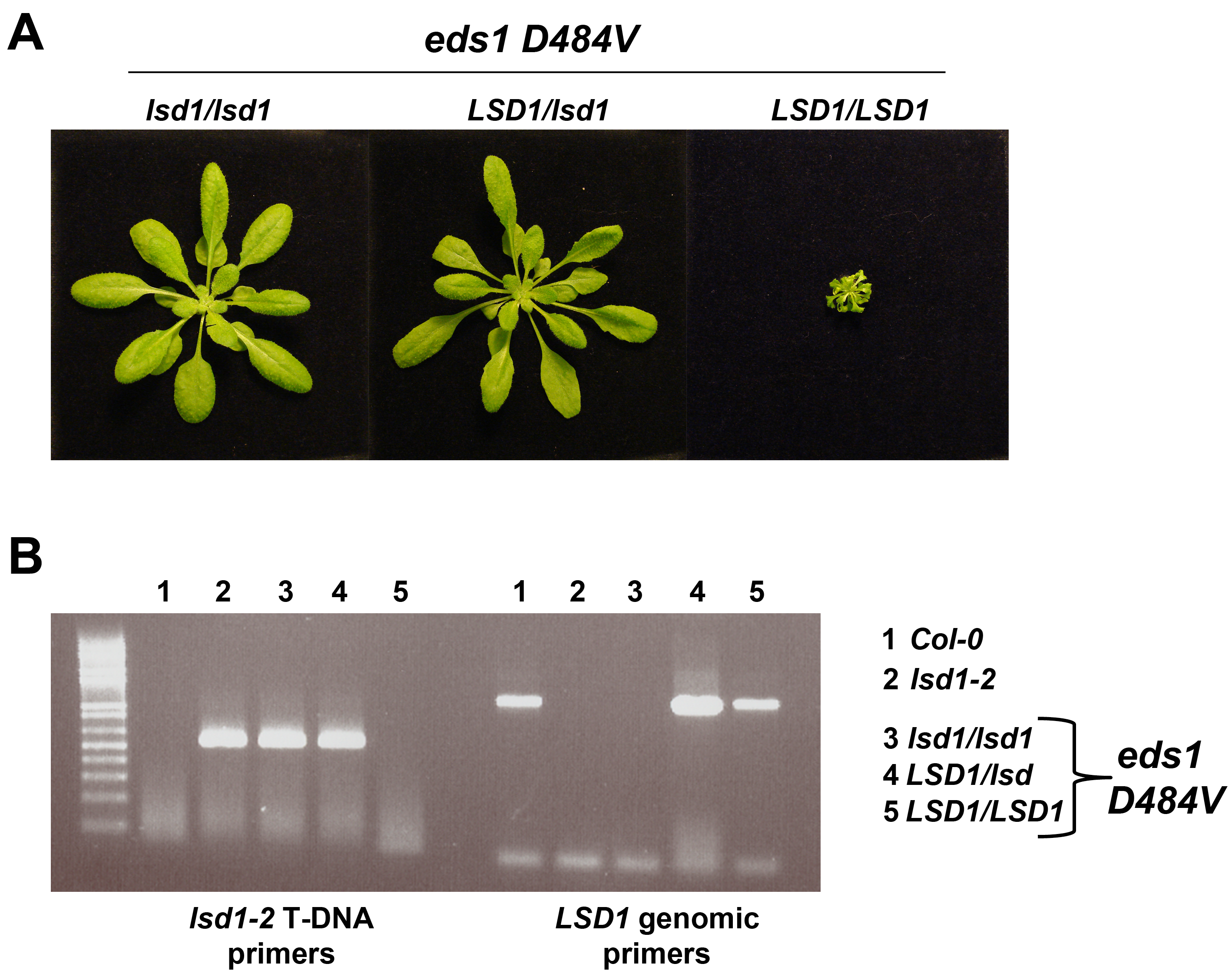

Supplement: Figure S2 — eds1 ADR1-L2D484V plants segregating LSD1 show both wild-type and extreme cpr phenotypes. (A) Pictures of plants homozygous for eds1 and ADR1-L2D484V and segregating lsd1. (B) PCR genotyping of the indicated genotypes confirms that only LSD1 homozygous eds1 ADR1-L2D484V (#5) plants have the severely stunted growth phenotype. #1 and 2 indicate the Col-0 and lsd1-2 controls respectively, #3–5 represent the genotypes from (A). (TIF) [file pgen.1003465.s002.tif]

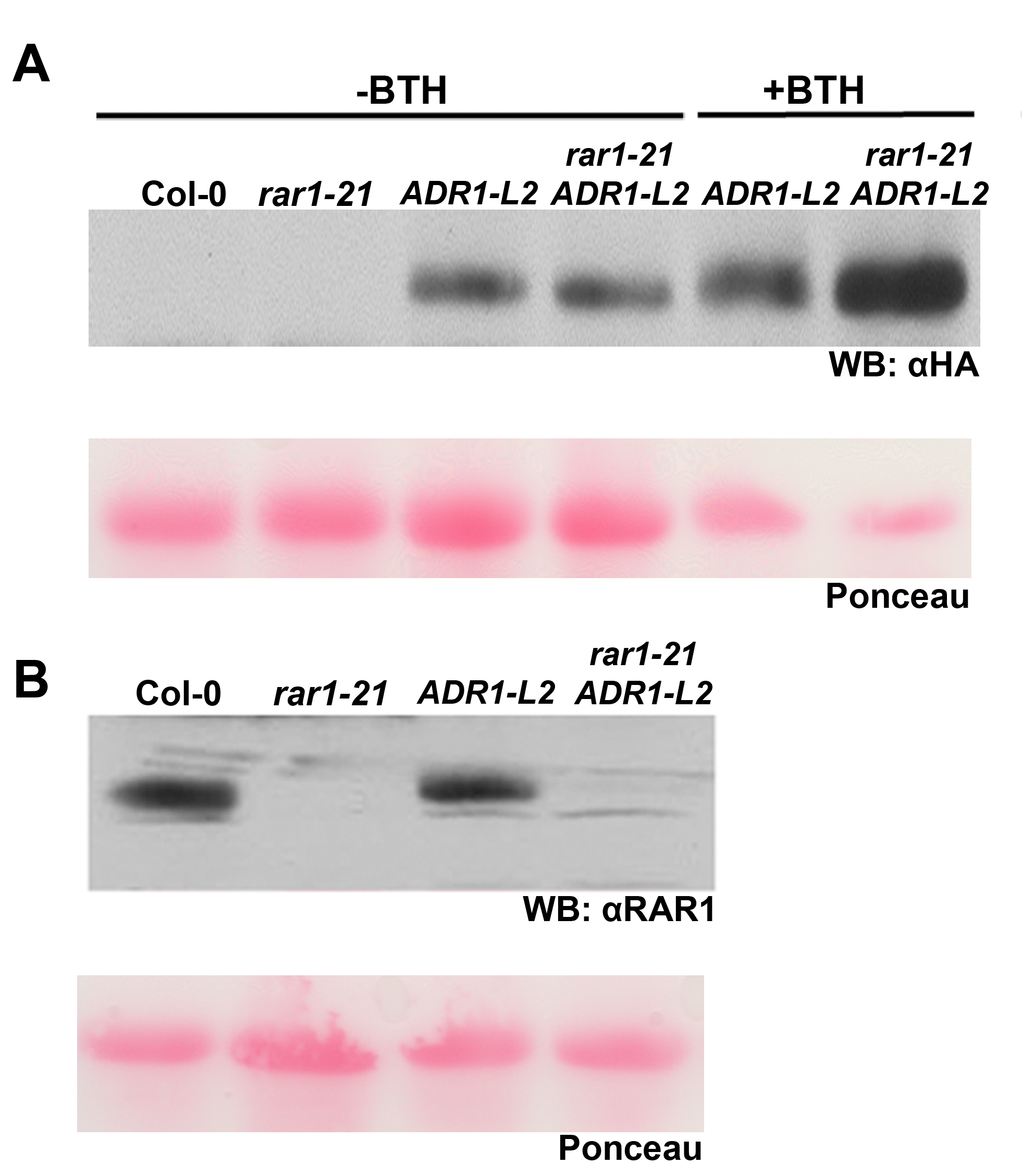

Supplement: Figure S3 — RAR1 is not required for either steady state ADR1-L2 accumulation or BTH-mediated induction. (A) ADR1-L2-HA and rar1-21 ADR1-L2-HA plants were sprayed with 300 µM BTH. Plants were collected for protein extraction 24 hpi. Protein from Col-0, rar1-21, and ADR1-L2-HA and rar1-21 ADR1-L2-HA plants + and -BTH were run on denaturing gels and probed with anti-HA antibody. (B) Protein from plants in (A) was also used in an anti-RAR1 Western blot to confirm the rar1-21 genotype. Ponceau stained blots in (A) and (B) show relative loading. (TIF) [file pgen.1003465.s003.tif]

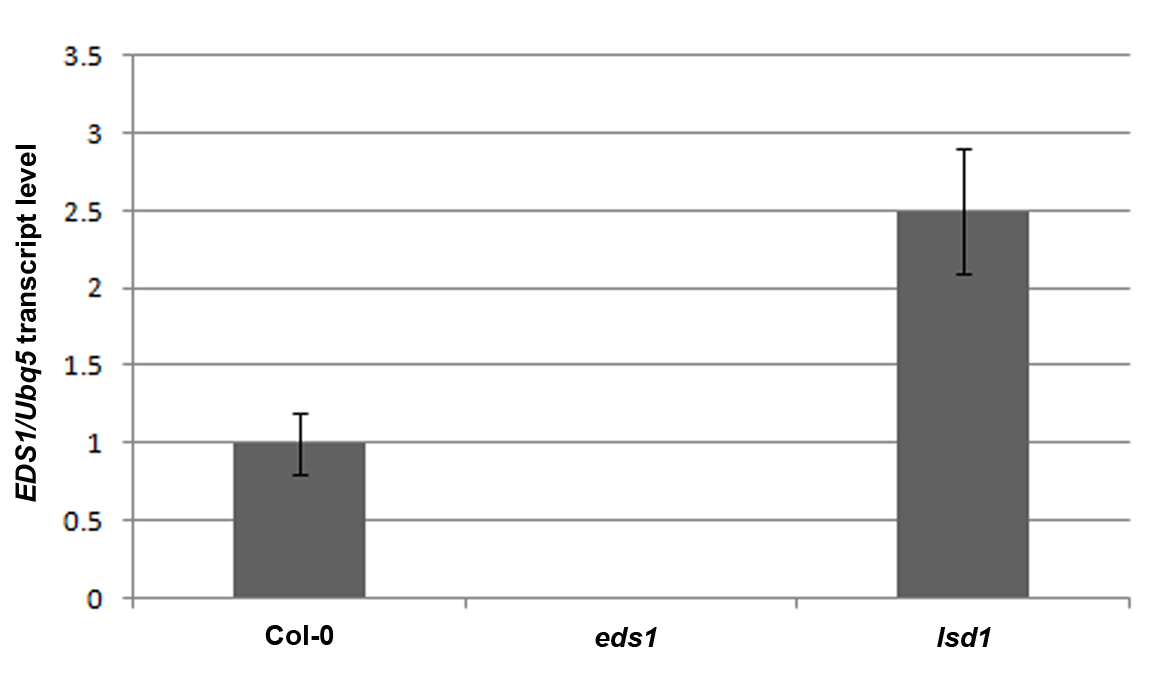

Supplement: Figure S4 — LSD1 negatively regulates EDS1 transcript. Quantitative real time PCR for the transcript amounts of EDS1 in Col-0, eds1-2, and lsd1-2. (TIF) [file pgen.1003465.s004.tif]
