# Supplementary material for: Genetic Requirements for Signaling from an Autoactive Plant NB-LRR Intracellular Innate Immune Receptor
Source: PLoS Genet. 2013 Apr 25;9(4):e1003465. doi: 10.1371/journal.pgen.1003465 (PMC3636237; doi:10.1371/journal.pgen.1003465)
Supplement: Table S1 — ADR1-L2D484V is lethal in an lsd1-2 background. Table of actual and expected genotypes of F3 progeny from a cross between lsd1-2 and ADR1-L2D484V shows that no lsd1-2 homozygous plants were recovered from plants that were homozygous for ADR1-L2D484V. ADR1-L2D484V was also transformed into lsd1-2, but no plants with a detectable amount of ADR1-L2D484V protein were recovered. (DOCX) [file pgen.1003465.s005.docx]

| Self cross of ADR1*-L2_D484V_* *lsd1* +/- | | |
| --- | --- | --- |
| Genotype | **Actual** | **Expected** |
| *LSD1/LSD1* | **50** | **31** |
| *LSD1/lsd1* | **74** | **62** |
| *lsd1/lsd1* | **0** | **31** |
| Total | **124** | **124** |
