# Supplementary material for: Genetic Requirements for Signaling from an Autoactive Plant NB-LRR Intracellular Innate Immune Receptor
Source: PLoS Genet. 2013 Apr 25;9(4):e1003465. doi: 10.1371/journal.pgen.1003465 (PMC3636237; doi:10.1371/journal.pgen.1003465)
Supplement: Table S2 — Primer sequences used in this work. (DOCX) [file pgen.1003465.s006.docx]

| Primer Name | Primer Sequence |
| --- | --- |
| For genotyping | |
| eds1-2F | AAGGCGTCTGTAGAGGAAAC |
| eds1-2R | CATATAGTCTCGCAGAGGAG |
| rar1-21F | TCACGACGGAATGAAAGAGTGGAGCTGCTACTAG |
| rar1-21R | TTTTGGAACCGATTTGGCCAGAACTGGTTTCTCAG |
| sid2-1F | AAGCTTGCAAGAGTGCAACA |
| sid2-1R | AAACAGCTGGAGTTGGATGC |
| AtMC1F | GCGTCACCTTCTCATCAACA |
| AtMC1R | ACGGTACCACTATGGCAAGC |
| LSD1F | CTGGGATTTGTAAAGCAGCTG |
| LSD1R | TCAAGTTCCATGGAGCAAAAG |
| ADR1-L2F | TTCTTACTGTGTGTCCCCAG |
| ADR1-L2R | CCTTCCTATCAATCCGATCG |
| For quantitative PCR analysis | |
| EDS1F | GACGGGGAAGTAGATGAGAAG |
| EDS1R | TCATCCATCATACGCTCACG |
| ADR1F | ATGGCTTCGTTCATAGATCTTTTC |
| ADR1R | CACATTGTAGGTGGTTCTAGG |
| ADR1-L1F | AAACCACTCTTGCCAAAGAAC |
| ADR1-L1R | GGATTTCCAGCTTCACAACC |
| ADR1-L2F | CCTCTTGATGTTCTCATCAAC |
| ADR1-L2R | GTAGCTAGTGTACATATGTCC |
